# Cutting-Edge PCN-ZnO Nanocomposites with Experimental and DFT Insights into Enhanced Hydrogen Evolution Reaction

**DOI:** 10.1021/acsaem.4c01932

**Published:** 2024-10-11

**Authors:** Narayan N. Som, Agnieszka Opalinska, Madhurya Chandel, Pratik M. Pataniya, Iwona Koltsov, Julita Smalc-Koziorowska, Anna Swiderska-Sroda, Stanislaw Gierlotka, Sumesh CK, Witold Lojkowski

**Affiliations:** 1 Institute of High Pressure Physics, Polish Academy of Science, Sokolowska 29/37, Warsaw 01-142, Poland; 2 Faculty of Mechatronics, Warsaw University of Technology, św. Andrzeja Boboli 8, Warsaw 02-525, Poland; 3 Department of Physical Sciences, P. D. Patel Institute of Applied Sciences, CHARUSAT, Changa, Gujarat 388421, India; 4 Łukasiewicz Research Network, Institute of Electrical Engineering, Warszawa 04-703, Poland

**Keywords:** PCN-ZnO nanocomposite, hydrogen evolution reaction, bifunctional electrocatalyst, alkaline electrolyte, density functional theory, solar-to-hydrogen efficiency

## Abstract

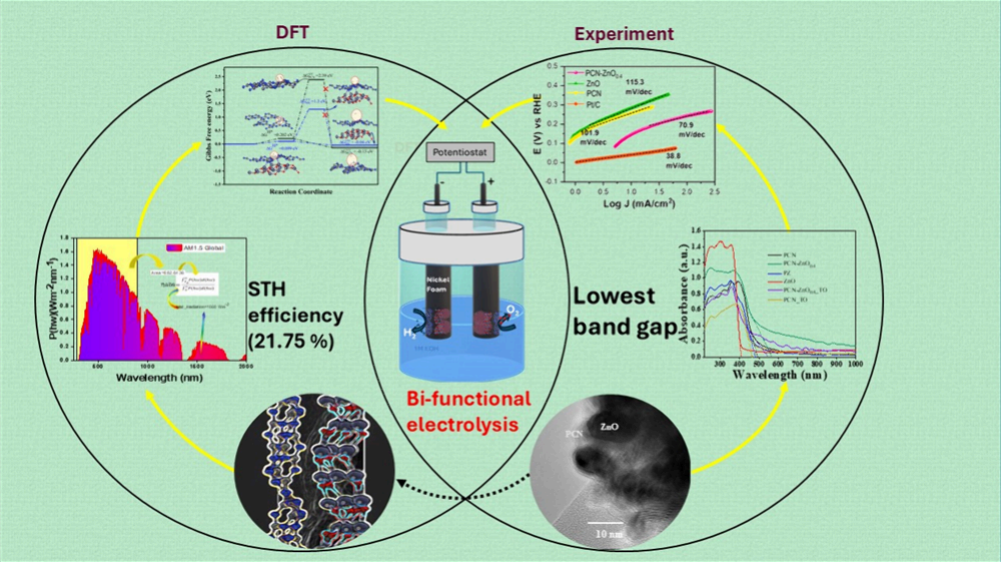

Polymeric carbon nitride (PCN) and PCN-ZnO nanocomposites are promising candidates for catalysis, particularly for hydrogen evolution reactions (HER). However, their catalytic efficiency requires enhancement to fully realize their potential. This study aims to improve the HER performance of PCN by synthesizing PCN-ZnO nanocomposites using melamine as a precursor. Two synthesis methods were employed: thermal condensation (Method 1) and liquid exfoliation (Method 2). Method 1 resulted in a composite with a 2.44 eV energy gap and reduced particle size, with significantly enhanced performance as a bifunctional electrocatalyst for simultaneous hydrogen and oxygen production. In contrast, Method 2 produced a nanocomposite with an enhanced surface area and a minor alteration in the band gap. In alkaline electrolytes, the PCN-ZnO_0.4_ nanocomposite synthesized with Method 1 exhibited high HER performance with an overpotential of 281 mV, outperforming pristine PCN (382 mV) and ZnO (302 mV), along with improved oxygen evolution reaction (OER) activity. Further analysis in a two-electrode alkaline electrolyzer using PCN-ZnO_0.4_ nanocomposite as both the anode and cathode demonstrated its promise as a bifunctional electrocatalyst. Density functional theory (DFT) calculations explained the enhanced catalytic activity of the PCN-ZnO nanocomposite, confirming that hydrogen evolution occurs through the Heyrovsky process, consistent with experimental results. Notably, the solar-to-hydrogen (STH) efficiency of the PCN-ZnO nanocomposite was four times greater, at 21.7% compared to 5.2% for the PCN monolayer, underscoring its potential for efficient solar-driven hydrogen production. This work paves the way for future advancements in the design of high-performance electrocatalysts for sustainable energy applications.

## 1 Introduction

Polymeric carbon nitride (PCN) is a promising candidate for photocatalyst and electrocatalyst application due to its nontoxic nature and cost-effectiveness in synthesis.^1,2^ Extensive efforts have focused on optimizing synthesis routes and precursor selection to enhance its catalytic activity for the hydrogen evolution reaction (HER) and oxygen evolution reaction (OER), with the goal of developing bifunctional electrodes for use in alkaline media.^3^ These efforts are driven by the urgent need to advance sustainable and renewable energy solutions. Major drawbacks of PCN as catalysts are the lack of active sites, high recombination rate, low charge carrier transfer, low conductivity, low surface area, and low solar-to-hydrogen efficiency for hydrogen production.^4−10^ Over the past decade, PCN nanocomposites have gained significant attention for their photocatalyst performance in pollutant degradation and water splitting.^4−10^

The efficiency of the nanocomposite (NC) made of the PCN and ZnO nanoparticles increased by about three to five times more than the PCN for the hydrogen evolution reaction (HER).^8,11−13^ The properties of PCN and its nanocomposites, such as surface area, band gap, and recombination rate, depend on the precursor and synthesis process for both PCN and ZnO.^4,8,14^ This can be illustrated by the following studies: In reference (14), PCN-ZnO NC synthesized using urea and a 40% mass ratio of ZnO exhibits a higher surface area of about 166 m^2^/g compared to PCN (74 m^2^/g) and a band gap of the 2.62 eV. Yu et al. reported that the PCN-ZnO NC synthesized using urea at a 10% relative weight and zinc hexahydrate exhibited a lower surface area of 12.8 m^2^/g compared to PCN’s 48.5 m^2^/g, along with a stronger light absorption in both the visible and ultraviolet region.^4^ In another study, Zhang et al. examined the properties of the PCN synthesized with different precursors, such as thiourea, dicyandiamide, melamine, and urea. The PCN obtained from melamine had a low surface area and low band gap compared to urea.^20^ Ma and Wang used melamine as a precursor for the PCN-ZnO NC and observed a higher surface area (34.4 m^2^/g) compared to that of PCN (5.87 m^2^/g). However, the band gap is slightly higher than pristine PCN. They reported 4.6 times higher hydrogen production than pristine PCN.^12^ Recently, Girish et al. reported that the PCN-ZnO NC exhibited a higher band gap (3.2 eV) compared to pristine PCN (2.96 eV) and a surface area of 48 m^2^/g, with three times higher hydrogen production compared to pristine PCN.^8^

The reported PCN photocatalysts and PCN-ZnO nanocomposites (NCs) still exhibit a band gap between 2.6 and 3.2 eV, limiting its ability to convert solar energy into hydrogen, achieving only about 10% solar-to-hydrogen conversion efficiency.^4,8,14^ To enhance efficiency, researchers are focusing on narrowing the band gap and improving other properties such as the nanocomposite’s surface area, nanoparticle size, charge carrier mobility, the heterointerface between materials, among many more factors that promote the catalytic kinetics.^15−19^ These combined optimizations are necessary for boosting the hydrogen evolution reaction.

We investigated whether using melamine as a precursor in the synthesis of PCN-ZnO NC would yield better results for the hydrogen evolution reaction and oxygen evolution reaction in an alkaline medium. Additionally, we analyzed the physical and optical properties of the PCN-ZnO nanocomposites to understand how the synthesis route affects their fundamental characteristics. Following this, density functional theory (DFT) calculations were used to understand the photocatalytic activity of both pristine PCN and PCN-ZnO nanocomposite through the calculation of adsorption energy, Gibbs free energy, overpotential of hydrogen evolution reaction (HER) and oxygen evolution reaction (OER), and solar-to-hydrogen efficiency (STH). DFT plays a crucial role in elucidating reaction mechanisms for complex processes such as water splitting, CO_2_ reduction reaction (CO_2_RR), methanol oxidation, and many others, providing insight into reaction pathways, energy barriers, and catalytic performance at the atomic level.^21−26^ Therefore, we employed the computational hydrogen electrode (CHE) approach to analyze the Volmer–Heyrovsky and Volmer–Tafel reactions to understand the HER mechanism.

## 2 Experimental and Computational Section

### Method 1: Synthesis of the PCN-ZnO Nanocomposite through Thermal Condensation

To synthesize the PCN-ZnO nanocomposite, we mixed the prepared ZnO and melamine. Specifically, we combined 20 weight percentages (wt %) ZnO with 80 wt % melamine by shaking them together in a plastic container. The mixture was then transferred to an alumina crucible and calcined at 550 °C for 3 hours (h), with a heating rate of 5 °C/min, in a muffle furnace in the air. This process resulted in the formation of the PCN-ZnO nanocomposite, referred to as PCN-ZnO_0.2._

Subsequently, PCN-ZnO nanocomposites with varying weight percentages (wt %) of ZnO, ranging from 20 to 80 (PCN/ZnO_*x*_, where *x* = 0.2, 0.3, 0.4, 0.5, 0.6, 0.7, and 0.8; see the Supporting Information), were synthesized using the same technique. Additionally, thermally oxidized etching of the PCN and PCN-ZnO_0.4_ was performed in the air at 550 °C for 2 h, resulting in PCN_TO and PCN-ZnO_0.4__TO, respectively.

### Method 2: Synthesis of the PCN-ZnO Nanocomposite Using Liquid Exfoliation

**Figure 1:**
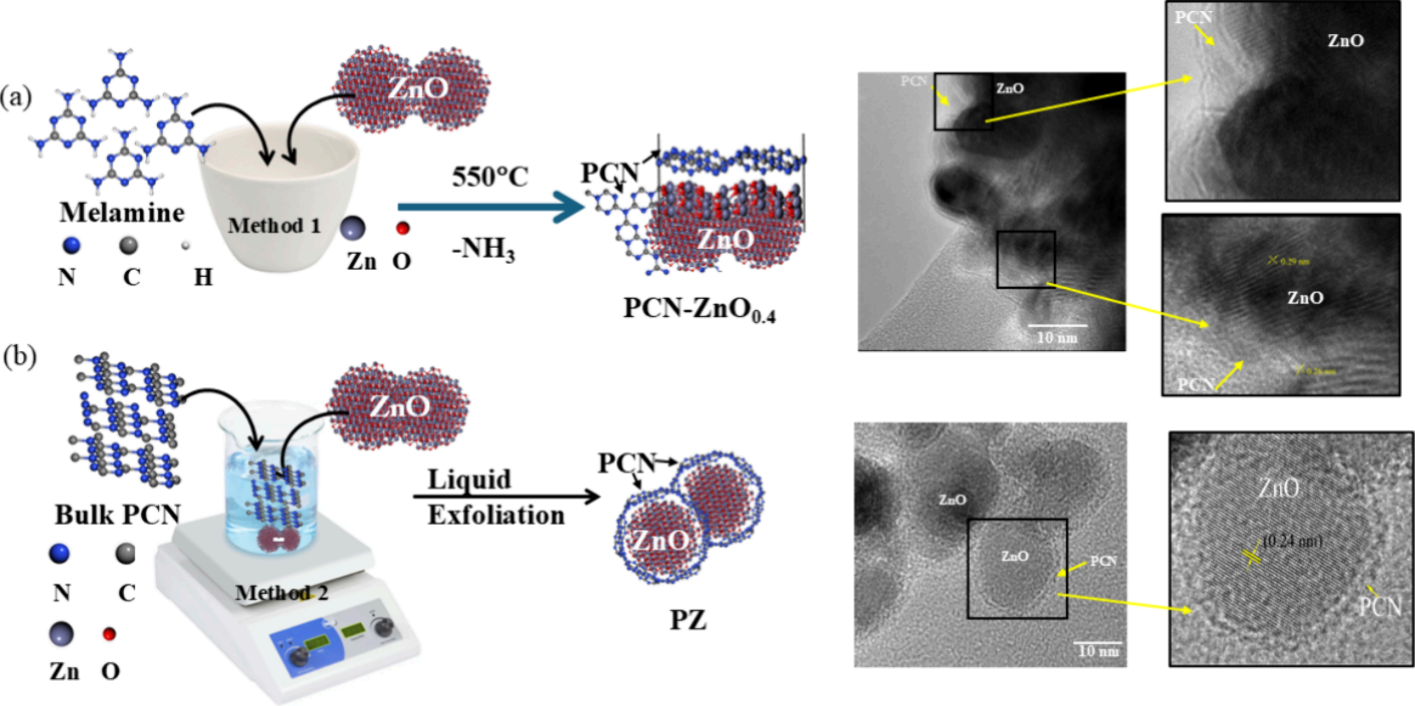
Schematic illustration of the synthesis of PCN nanocomposite—(a) Method 1 and (b) Method 2—along with their corresponding HRTEM image as a reference.

Freshly calcined PCN was dissolved in ethanol with vigorous stirring at a constant temperature of 70 °C and a stirring speed of 250 rpm, followed by adding 40 wt % of ZnO. The mixture was then left to stand for a day. Subsequently, the resulting mixture was dried in air at 160 °C for 3 h and designated as PZ. The synthesis process of PCN and ZnO nanoparticles, along with the characterization sections, is available in the Supporting Information. A schematic of the synthesis route is shown in Figure 1, alongside TEM images of the nanocomposite.

#### Electrochemical Measurements

The hydrogen and oxygen evolution reactions on the prepared ZnO, PCN, and PCN-ZnO_0.4_ catalysts were measured using a standard three-electrode setup with a Metrohm PGSTAT-M204 workstation. For the preparation of working electrodes, 20 mg of as-prepared catalysts were dispersed in 1 mL ethanol with 10 μL of Nafion binder. The suspension was sonicated for 1 h to prepare the catalyst ink. Meanwhile, the nickel foam was cleaned using acetone, distilled water, and a 2 M HCl solution to remove impurities and the oxide layer. Then, two drops of 10 μL of catalyst ink were decorated on the 1 × 1 cm^2^ area of the pretreated nickel foam. The electrodes were then dried at 70 °C for 12 h in a vacuum. Ag/AgCl (3 M KCl saturated) and graphite rod were used as the reference and counter electrodes, respectively. The electrochemical measurements were performed in an alkaline electrolyte (1 M KOH). First, the electrodes were analyzed by linear sweep voltammetry (LSV) curves recorded at a scan rate of 2 mV/s. The polarization curves were measured for HER and OER performance with *iR* compensation. Tafel plots were also calculated to investigate reaction kinetics. Electrochemical impedance spectra (ESI) were recorded in the frequency range of 10 mHz to 100 kHz to study the charge transfer and interfacial resistance. The cyclic-voltammetry (CV) curves were recorded at different scan rates in the nonfaradic potential range, and double-layer capacitance (Cdl) was calculated.

#### Computational Details

All the density functional theory (DFT) calculations were carried out using the norm-conserving pseudopotential with the Quantum Espresso code.^27^ The exchange-correlation interaction was treated using the generalized gradient approximation (GGA) proposed by Perdew–Burke–Ernzerhof (PBE),^28^ while long-range van der Waals interactions were accounted for by including Grimme’s dispersion correction (D2) to void underestimating the adsorption energy.^29^ A dense Monkhorst–Pack grid of 7 × 7 × 1 was used for sampling the reciprocal space.^30^ The Marzari–Vanderbilt smearing method was applied, with the energy convergence achieved at a threshold of 10^–4^ eV between consecutive steps. The convergence process was repeated self-consistently until the maximum Hellmann–Feynman forces acting on each atom were less than 0.001 eV/Å. An iterative Davidson-type diagonalization approach was used to solve Kohn–Sham equation, achieving an energy convergence threshold of 1 × 10^–10^ Ry.

## 3 Results and Discussion

### 3.1 Phase Composition Analysis

**Figure 2:**
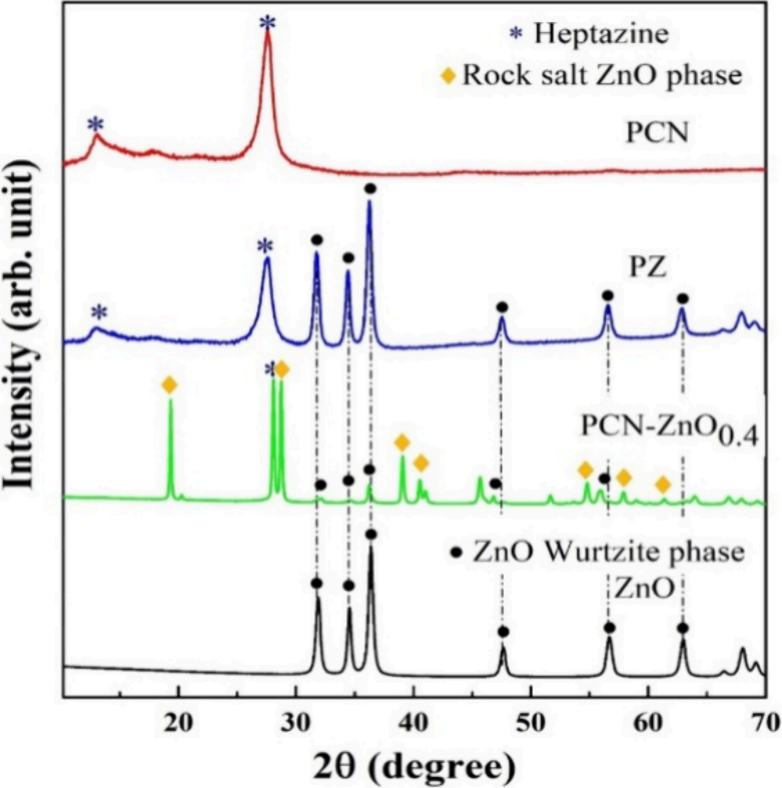
XRD pattern of ZnO, PCN-ZnO_0.4_ nanocomposite (Method 1), PZ nanocomposite (Method 2), and PCN.

The PCN, ZnO, and its nanocomposite phase structure were analyzed by using XRD, as shown in Figure 2. PCN exhibits two major peaks: a low-intensity peak at 13° corresponding to the (100) plane and a strong peak at 27.3° associated with the (200) plane, consistent with previous reports.^1,10,31^ The observed diffraction peaks of pure ZnO are 31.92, 34.56, 36.39, 47.67, 56.73, and 63.0° corresponding to the planes are 100, 002, 101, 102, 110, and l03, respectively. These peaks are consistent with the wurtzite hexagonal crystal phase (JCPDS card no. 36-1451), aligning with the previous synthesis process.^4,8,10,32,33^ The average crystallite sizes of ZnO and PCN using Debye–Scherrer’s equations are 23.7 and 13.6 nm, respectively (Table 1). We analyzed the diffraction peak of the nanocomposite PCN/ZnO_*x*_ (*x* = 0.2 to 0.8) obtained from Method 1, as shown in Figure S1a. We observed the coexistence of the rock salt (RS) ZnO phase^32,33^ (JPCDS no. 21-1486) consisting of the diffraction peak at 19.5° reported for Zn(CH_3_COO)_2_,^34^ while the diffraction peaks at 39.0, 40.6, 54.7, 57.7, and 61.65° were reported for the rock salt ZnO phase.^32^ In Method 1, the diffraction peaks of RS ZnO phase are observed up to 50 wt % of ZnO concentrations. Beyond that, prominent peaks of both the PCN and RS ZnO phases disappeared (Figure S1a). A possible explanation could be the absence of PCN formation (with ZnO concentrations exceeding 50 wt %) and the limited interaction between melamine and ZnO. As a result, the wurtzite phase does not transform to the RS ZnO phase, and thus, no prominent peak of the RS ZnO phase is detected. Moreover, we observed an increase in the crystallite size of nanoparticles, which is mentioned in Table S1. We have compared the XRD peaks of PCN-ZnO_0.4_ (Method 1), PZ (Method 2) (Figure 2), and PCN/ZnO_0.4__TO (thermal oxidation etching), as shown in Figure S1b. The XRD pattern of PZ exhibits diffraction peaks of heptazine (PCN) and the wurtzite ZnO phase. In PCN-ZnO_0.4_, the diffraction peak of heptazine (27.9° of PCN) dominates over both the Wurtzite and the RS ZnO phases. This indicates that the individual phases of ZnO and PCN are present in the composite. In other words, ZnO and PCN maintain their crystalline structures by forming a new combined lattice structure in the composite material. Interestingly, after the thermal oxidation etching of PCN-ZnO_0.4_ (i.e., PCN/ZnO_0.4__TO), we observed a more intense diffraction peak corresponding to the wurtzite ZnO phase, while the intensity of the RS ZnO phase decreased. This suggests that the wurtzite phase becomes more dominant following thermal oxidation etching (TO), which could be attributed to the transformation of the RS ZnO phase into the wurtzite phase (Figure S1b). In the case of PCN_TO, we observed that the intensity of the 002 plane of PCN increased after thermal oxidation etching, leading to a slightly lower crystallite size compared to pristine PCN (Table 1).

**Table 1:** Highest Intensity Peak’s 2θ Value (Degree), Average Crystallite Size (*D*/nm), Density (g/cm^3^), Specific Surface Area (m^2^/g), Sauter Mean Diameter (SMD/nm), and Direct Band Gap (*E*_g_/eV) of PCN, ZnO, PCN-ZnO_0.4_, PZ, PCN-ZnO_0.4__TO, and PCN_TO

prepared samples	2θ (degree)	*D* (nm)	density (g/cm^3^)	SSA_BET_ m^2^/g	SMD (nm)	*E*_g_ (eV)
PCN	27.78	13.67	1.73	10.27	33.77	2.67
ZnO	36.39	23.66	5.24	45.48	25.17	3.11
PCN-ZnO_0.4_	28.77	21.62	2.84	3.12	677.13	2.44
PZ	36.27	20.43	2.14	15.12	185.43	2.74
PCN-ZnO_0.4__TO	36.36	30.32	2.76	10.46	207.83	2.87
PCN_TO	27.9	13.614	1.82	60.01	54.94	2.97

In summary, PZ shows a slightly smaller crystallite size compared to PCN-ZnO_0.4_. These observations confirm the successful formation of the nanocomposites PCN-ZnO_0.4_ and PZ. The thermal oxidation etching of PCN-ZnO_0.4_ leads to the growth of ZnO nanoparticles. After this process, the intensity of crystallite plane wurtzite ZnO phase increased compared to PCN-ZnO_0.4_, and it exhibits a larger crystallite size compared to the other two nanocomposites. Furthermore, the intensity of the 002 plane of PCN decreased after thermal oxidation etching, which may be attributed to the evaporation of PCN into constitute gases.

### 3.2 SEM, EDS, and TEM Analysis

**Figure 3:**
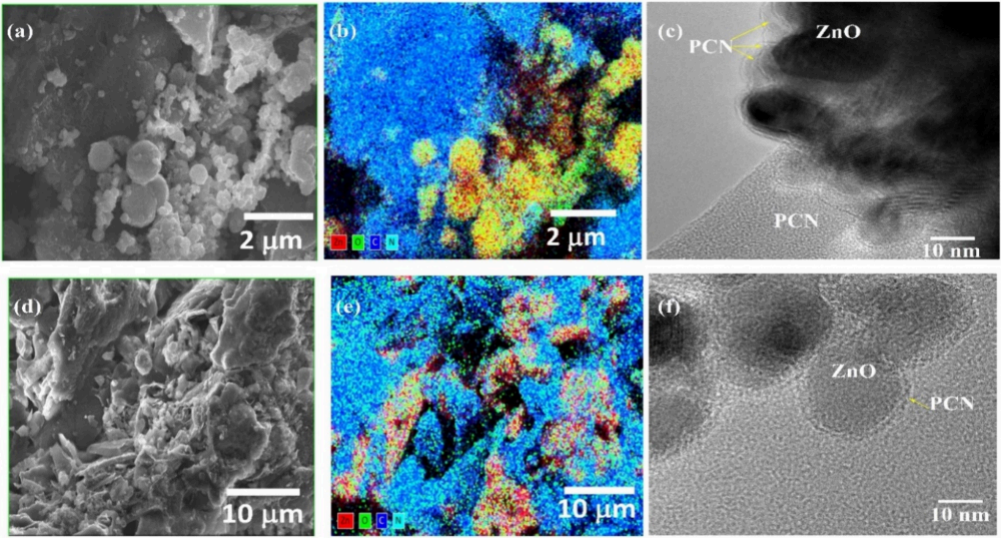
SEM (a, d), EDS mapping (b, e), and HRTEM image (c, f) of PCN-ZnO_0.4_ (top panel) and PZ (bottom panel), respectively.

We have conducted SEM and TEM analyses to understand the nanocomposite morphology. We observed that ZnO forms an agglomeration of small particles, whereas PCN had a rough surface emerging in a lamellar shape, as shown in Figure S2. PCN-ZnO_0.2_ and PCN-ZnO_0.3_ resemble PCN with an agglomeration of tiny particles of ZnO leading to block-shape formation. In Figure S2a, the spherical agglomeration of ZnO and PCN, along with a dense, smooth surface of PCN, was visible. We observed the pure and bigger crystallite formation of ZnO after the concentration of ZnO increased beyond 50 wt %. This can be seen in Figure S2, whereas in PCN-ZnO_0.8_, the wurtzite phase gets agglomerated along with the more extensive RS ZnO phase. In the case of PZ, lamellar formation of PCN with an agglomeration of ZnO nanoparticles was observed. In Figure 3b,e, the elemental mapping of PCN-ZnO_0.4_ and PZ is shown, respectively. It differentiated the lamellar PCN and agglomerate ZnO nanoparticles. The yellow region is ZnO in both cases.

We can see denser blue and cyan regions in the case of PCN-ZnO_0.4_ compared to PZ. It forms a thinner lamellar PCN due to liquid exfoliation in PZ. The TEM image can further confirm this, as shown in Figure 3c,f. We have observed a denser PCN ring layer formation near ZnO nanoparticles in PCN-ZnO_0.4_, whereas there was a lighter ring formation near ZnO nanoparticles in PZ. We calculated the *d*-spacings of the lattice fringes for PCN-ZnO_0_._4_ NC, which are approximately 0.26 and 0.29 nm, corresponding to the (002) and (100) planes of the wurtzite ZnO phase, respectively (Figure 1; zoomed-in section of the HR-TEM of PCN-ZnO_0_._4_ NC). In contrast, for PZ NC, the observed *d*-spacing is about 0.24 nm, attributed to the (101) plane of the wurtzite phase, consistent with the XRD pattern. We also calculated the elemental atomic percentage of the nanoparticles ZnO and PCN and their nanocomposites (PZ and PCN-ZnO_0.4_), as shown in Table S3. In the wurtzite ZnO phase, the atomic percentage (at. %) of zinc and oxygen is about 48.54 and 51.46 at. %, respectively. The PCN exhibits C and N atomic percentages of about 39.10 and 57.38 at. %, respectively. To evaluate the presence of PCN, we calculate the C/N ratio, which is 0.68 in the case of pristine PCN. In Method 1, the content of PCN is highest at 20 wt %, and it decreases with the concentration of ZnO. The C/N ratio is about 0.74, 0.81, and 0.93 for PCN-ZnO_0.2_, PCN-ZnO_0.3_, and PCN-ZnO_0.4,_ respectively. The highest C/N ratio is that for PCN-ZnO_0.4_. The ratio is much higher in the case of PZ, which confirms the formation of the thin layer, which removes nitrogen during the nanocomposites’ drying process.

### 3.3 Thermal Analysis Coupled with Evolved Gas Analysis

The thermal stability of synthesized samples ZnO, PCN, PCN-ZnO_0.4_, and PZ was investigated using simultaneous thermal analysis (STA), i.e., thermogravimetric analysis (TGA) and differential scanning calorimetry (DSC) methods at the same time. Gases evolved from the samples during heating were analyzed in situ using mass spectrometry (MS) method. The samples were heated up to 1000 °C at a rate of 10 °C/min in a helium atmosphere. The ZnO (Figure S3a) nanoparticles are stable up to 1000 °C, with a mass loss of about 2.5%. The DSC curve shows exothermic effects, corresponding to the release of CO_2_ (*m/z* = 44) with maxima of MS signals at approximately 300, 450, and 670 °C. An increase of H_2_O (*m/z* = 18) signal was also detected with maxima at about 50 and 380 °C (Figure S3a). PCN undergoes complete decomposition in the temperature range of 600–780 °C, with emission of nitrogen oxides/ammonia (NO_2_/NH_3_, *m/z* = 16), hydrazine/methanol (H_2_N-NH_2_/CH_3_OH, *m/z* = 32), cyanogen ((CN)_2_, *m/z* = 52), ethylene (C_2_H_4_, *m/z* = 28), and cyanide/hydrogen cyanide (CN-/HCN, *m/z* = 27) (Figure S3b).^2^

For the PCN-ZnO_0.4_ composite, the total mass loss heated up to 1000 °C is about 26%. TGA-DSC analysis reveals an endothermic effect with the extreme at 100 °C, likely due to water release and transition from the RS ZnO phase to the wurtzite type. This can be confirmed by XRD results (see Figure S1) showing that the thermal oxidation etching of PCN-ZnO_0.4_ shows low-intensity XRD peaks of the RS ZnO phase, confirming its transition to the wurtzite phase. Intensive mass loss in the temperature range of 650 and 700 °C occurs due to the removal of H_2_O, CO_2_, NO_2_/NH_3_, H_2_N-NH_2_/CH_3_OH (*m/z* = 32), cyanogen ((CN)_2_, *m/z* = 52), C_2_H_4_ (*m/z* = 28), and CN-/HCN (*m/z* = 27). Additionally, the MS signal of a new radical, butadiene [C_4_H_6_]^+^, is detected at the temperature range 700–750 °C. In summary, the mass loss of ZnO and PCN-ZnO_0.4_ heated to 1000 °C is 2.5 and 26%, respectively, indicating that the amount of PCN in the composite is about 23.5%.

In the PZ nanocomposite, PCN fully decomposes at around 780 °C, releasing all the aforementioned gases except butadiene [C_4_H_6_]^+^, resulting in a mass loss of 77.21%, and leaving behind ZnO. The same gases are released in PCN_TO, with full decomposition at nearly 750 °C and a total mass loss of about 98.75%. PCN-ZnO_0.4__TO shows a loss pattern similar to PCN-ZnO_0.4_, including traces of butadiene [C_4_H_6_]^+^, with total mass losses of 12.5% at 675 °C, 25% at 750 °C, 30% at 850 °C, and 43.75% at 1000 °C. Overall, the presence of various (RS) ZnO phases in Method 1 PCN-ZnO_0.4_ NC uniquely enhances the stability of the nanocomposites. Additionally, it reduces the optical band gap of PCN-ZnO_0.4_ compared to PZ, showcasing a novel approach to improving the material’s properties.

### 3.4 Specific Surface Analysis (SSA_BET_)

Surface area is one of the significant factors in nanocomposites and plays an essential role in hydrogen production. Therefore, Brunauer–Emmett–Teller (SSA_BET_) analysis was performed. We have calculated the nanoparticles’ Sauter mean diameter (SMD) by using the SSA_BET_ and the bulk density of the particulate material with the assumption of spherical particles. The specific surface area, density, and SMD are given in Table 1. The specific surface areas of ZnO and PCN were about 10.27 and 45.48 g/m^2^_,_ respectively, which agree with the previous report.^9,35,36^ The nanocomposite obtained with Method 1 acquires a lower SSA_BET_ due to the thermal polymerization of melamine on the ZnO surface, leading to the larger microcrystal with high crystallinity and coexistence of the RS ZnO phase. In Figure S3, the growth of bigger-size microcrystal PCN-ZnO_0.7_ compared to PCN-ZnO_0.3_ can be seen. However, the PZ nanocomposite exhibits a higher SSA_BET_ due to the liquid exfoliation of the bulk PCN, which is confirmed by the TEM images (Figure 3c,f). We can see the formation of the dense layer of PCN near ZnO in the case of PCN-ZnO_0.4_, while in PZ, a thin layer forms in the PCN periphery of the ZnO nanoparticles due to the liquid exfoliation of PCN. We observed that the smaller the particle size, the larger the SSA_BET_. Moreover, the density of the Method 1-based nanocomposites is close to that of PCN, and with a higher concentration of the ZnO, the density is close to that of ZnO.

### 3.5 Chemical Analysis

Further, an analysis was conducted to investigate the interaction between PCN and ZnO and the interference in the formation of PCN from melamine. Attenuated total reflectance infrared (FTIR-ATR) analysis was performed, as this technique allows for the examination of molecular vibrations and interactions between different compounds by measuring the absorption of infrared radiation as it interacts with the sample surface.

**Figure 4:**
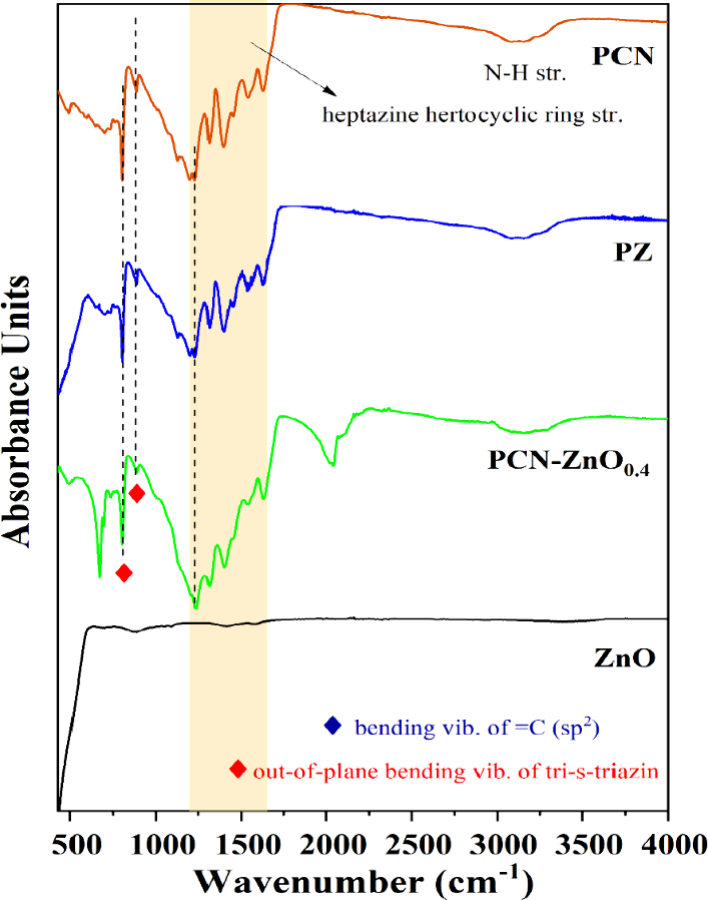
ATR-IR spectra of ZnO, PCN-ZnO_0.4_, PZ, and PCN.

The strong absorption peak in the range of 400–600 cm^–1^ is characteristic of Zn–O stretching vibrations (bottom panel of Figure 4), indicating the presence of the Zn–O bond in the ZnO lattice structure.^8^ The distinct absorbance peaks at 810 and 890 cm^–1^ and in the range of 1200–1600 cm^–1^ are related to the out-of-plane bending vibrations of tri-*s*-triazine, N–H deformation, and heterocyclic C–N bond, respectively.^1,20,31,37^ The absorbance peak in the 3300–3500 cm^–1^ range is related to N–H stretching. This confirms the formation of the polymeric carbon nitride. In Figure 4, PCN and ZnO absorbance in the PCN-ZnO nanocomposites confirmed its formation. In Figure S4a, we observed that the bands characteristic of PCN (at 810, 890, 1234, and 1630 cm^–1^) are present only when the amount of ZnO does not exceed 40 wt %. Moreover, we observed two additional peaks at 675 cm^–1^ and a hump near 2100 cm^–1^, which were also observed in the previous report.^32^ The peak at 675 cm^–1^ could also associate with the interaction between ZnO and PCN. This could lead to shifts in the vibrational modes compared to their pristine counterparts. The hump near 2100 cm^–1^, observed in the urea-based PCN-ZnO nanocomposite, which relates to the increment in the concentration of ZnO, leads to the breakage of the triazine unit with the formation of the C–N bond instead of sp^2^ bonds.^14^ We observed that the ZnO crystal size increases with increasing ZnO concentration, preventing of the RS-ZnO phase formation and leading to the dominance of the wurtzite phase. This also inhibits the thermal polymerization of melamine, which deteriorates the properties of heptazine rings.^14^ After the thermal oxidation etching, this phenomenon becomes more pronounced, as the intensity of bands (800 to 1634 cm^–1^) drastically decreased, while the intensity of the hump (near 2100 cm^–1^) increases (Figure S4b). The disappearance of N–H stretching also confirms the decomposition of PCN into constituent gases (Figure S3e). This can be attributed to the decomposition of PCN on the surface of ZnO. In PZ, no such peaks were observed, indicating the formation of stable PCN, and a slight decrement in the intensity of all of the major absorbance peaks due to the formation of the nanocomposites (Figure 4). A blue shift was observed in out-of-plane bending vibrations of tri-*s*-triazine at 803.84 and 804.31 cm^–1^ for PCN-ZnO_0.4_ and PZ, respectively. Interestingly, we observed a red shift (1234.44 cm^–1^) in the bending vibration of =C (sp^2^) for PCN-ZnO_0.4_ compared to PCN, which could be an increase in bond length. This confirms the strong interaction in PCN-ZnO_0.4,_ which alters the heptazine heteroring. Method 1 (PCN-ZnO_0.4_) demonstrates a strong interaction between ZnO and PCN due to the thermal polymerization of melamine into PCN on the ZnO surface. This interaction likely induces the formation of the RS ZnO phase, creating distinct scenarios not observed in Method 2.

### 3.6 Ultraviolet–Visible Spectroscopic Analysis

**Figure 5:**
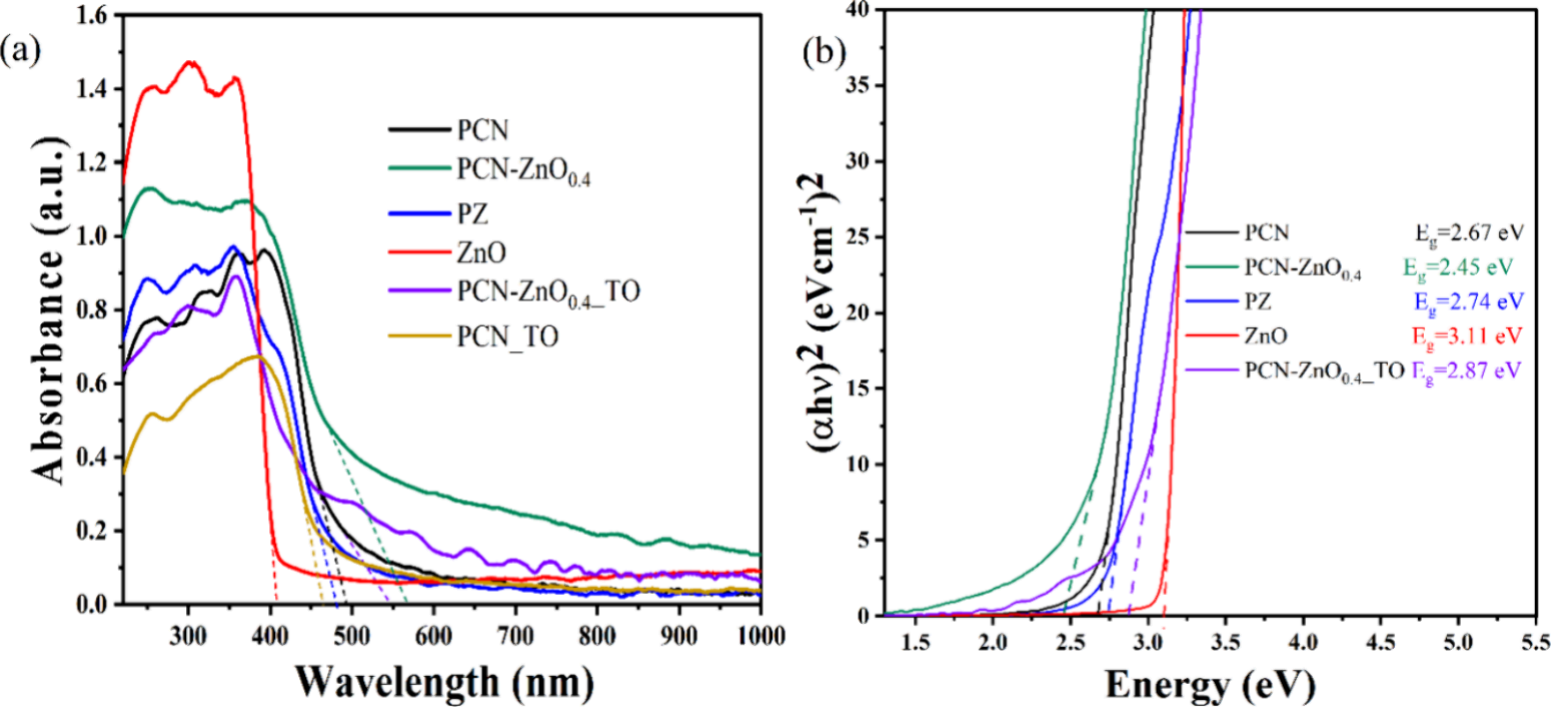
(a) Absorbance spectra and (b) Tauc plot of PCN, ZnO, and its nanocomposites (PCN-ZnO_0.4_, and PZ).

Further, the synthesized material’s optical properties have been measured using ultraviolet–visible (UV–vis) based diffused reflectance spectra (DRS). These optical properties are crucial for solar-based hydrogen production. PCN-ZnO_0.4_ exhibits a longer wavelength absorption edge than pristine ZnO and PCN, as shown in Figure 5a.

The sharp increase in light absorption occurs at 407, 480, and 566 nm for ZnO, PCN, and PCN-ZnO_0.4,_ respectively. Therefore, the intrinsic band gap based on the absorbance edge is 3.05, 2.67, and 2.19 eV, respectively. Comparing the absorbance edge, Method 1 shows a lower band, which may be related to the coexistence of the RS ZnO phase and stronger interactions. This resulted in the formation of defective states. In Method 2, the dissolution of PCN in ethanol may lead to fewer deposition layers on the surface of the ZnO phase. Previously, thermal oxidation etching of the PCN was shown to lead to the formation of a nanosheet and a shift in band gap to 2.97 eV.^38^ We also observed that after thermal oxidation etching of both PCN and PCN-ZnO_0.4_, the band gap was higher compared to that of their pristine counterparts. We used the Tauc plot method to determine the optical band gap. The band energies (*E*_g_) were estimated from the intercept of the tangent plot of (α*h*ν)^2^ vs photon energy for the direct band, which is shown in Figure 5b. PCN-ZnO_0.4_ shows the lowest optical direct band gap, i.e., 2.44 eV, and indirect band gap, i.e., 2.04 eV, as shown in Figure S4.

To our knowledge, we have uniquely synthesized a PCN-ZnO nanocomposite with a lower band gap using melamine, outperforming a comparable nanocomposite derived from urea thermal polymerization and ZnO synthesis. This reduction in the band gap of PCN is significantly influenced by the precursor used, the synthesis route, and the coexistence of impurities and additional phases within the nanocomposite.^20,31^ This reduction in the nanocomposite band gap could uniquely improve absorption in the visible region, thereby enhancing solar-to-hydrogen efficiency through the increased production of exciton pairs.^39^ For a deeper understanding, HER (hydrogen evolution reaction) and oxygen evolution reaction (OER) studies were conducted using advanced nanomaterials, including ZnO, PCN, and PCN-ZnO_0.4_.

### 3.7 Hydrogen and Oxygen Evolution Reaction in Alkaline Electrolyte

**Figure 6:**
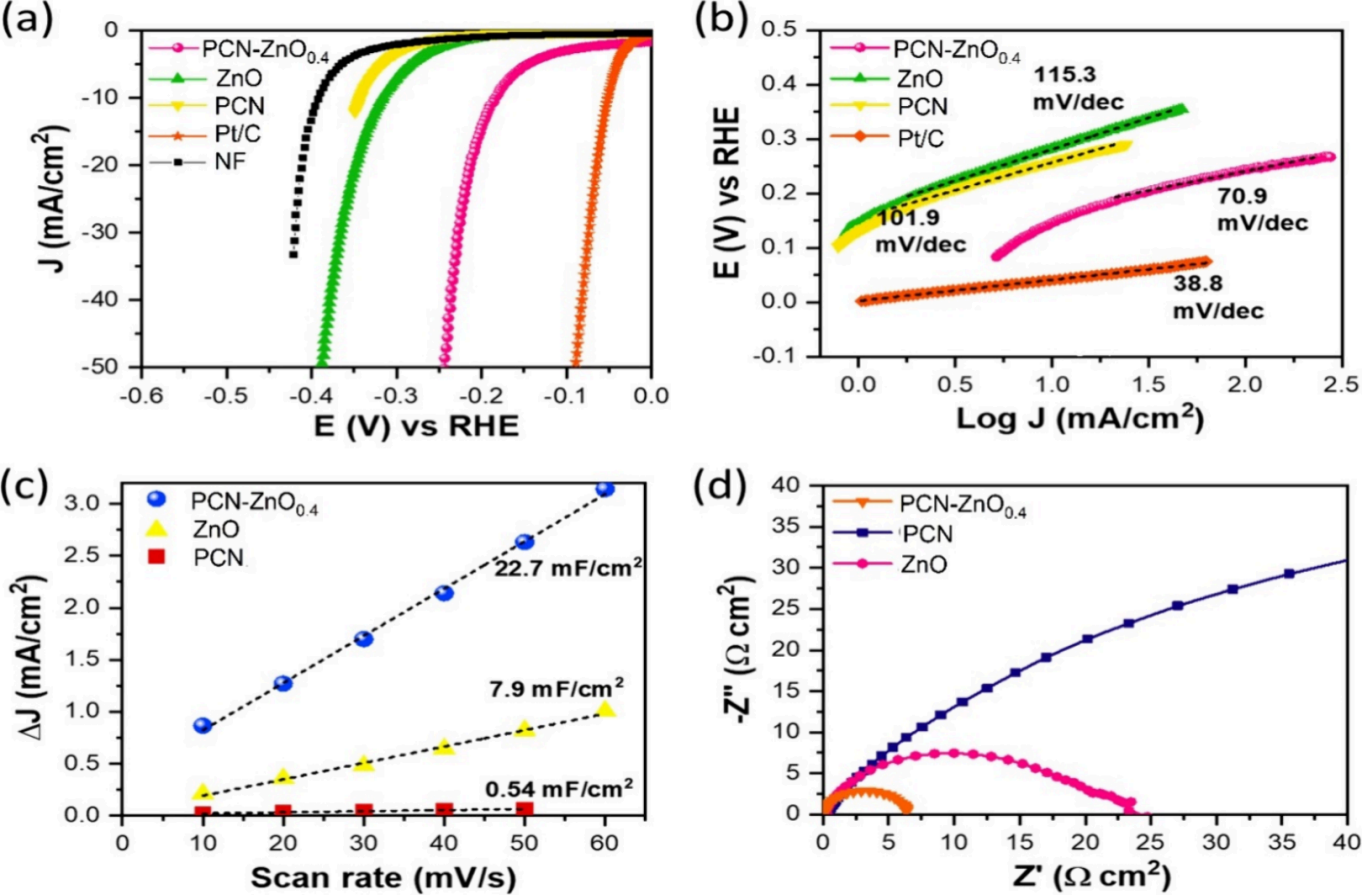
Hydrogen evolution reaction on ZnO, PCN, PCN-ZnO0.4, bare nickel foam (NF), and commercial Pt/C electrocatalysts in 1 M KOH. (a) Polarization curves recorded at a scan rate of 5 mV/s with *iR* compensation. (b) Tafel plots. (c) Double layer capacitance (Cdl) and (d) EIS curves at 100 mV vs RHE.

The electrocatalytic activity of electrodes based on ZnO, PCN, PCN-ZnO_0.4_, bare Ni-foam (NF), and commercial Pt/C was analyzed in a 1 M KOH water electrolyte. Figure 6a shows the polarization curve for the hydrogen evolution reaction in an alkaline medium, indicating that PCN-ZnO_0.4_ electrodes exhibit superior HER activity compared to control electrodes. According to the polarization curve, PCN-ZnO_0.4_ exhibits an overpotential of 186 mV to generate a geometric current of 10 mA cm^–2^. PCN-ZnO_0.4_ shows much lower overpotential as compared to pristine ZnO (316 mV@10 mA cm^–2^) and PCN (342 mV@10 mA cm^–2^), demonstrating the promotion role of the PCN-ZnO_0.4_ interface in HER activity.

The Tafel slope values further evaluate the HER kinetic. Figure 6b shows that the Tafel slope values are 115.3 mV/dec for ZnO and 101.9 mV/dec for PCN. The Tafel slope value is reduced to 70.9 mV/dec for PCN-ZnO_0.4_, indicating accelerated water dissociation kinetics with the Heyrovsky process as the rate-determining step. The higher Tafel slope value for ZnO and PCN shows that the water dissociation is challenging owing to sluggish charge transport.^40,41^

To analyze the role of the electrochemical surface area (ECSA), double-layer capacitance was evaluated using CV curves in nonfaradic regions. The significantly higher Cdl of the PCN-ZnO_0.4_ electrode (22.7 mF cm^–2^) compared to ZnO (7.9 mF cm^–2^) and PCN (0.54 mF cm^–2^) (Figure 6c) can be attributed to the formation of a heterointerface between PCN and ZnO, which enhances charge transfer and increases the electrochemically active surface area. Previously reported studies have shown that this increase in ECSA directly contributes to improved catalytic performance, as it provides more active sites for the hydrogen evolution reaction (HER) and oxygen evolution reaction (OER).^17,18^ In contrast, the control electrodes, ZnO and PCN, exhibit sluggish HER activity in alkaline electrolytes due to a lower concentration of active catalytic sites.^42,43^

Electrochemical impedance spectroscopy (Figure 6d) further reveals much lower charge transfer resistance for PCN-ZnO_0.4_ (6.3 Ω·cm^2^) compared to ZnO (23.3 Ω·cm^2^) and PCN (140.0 Ω·cm^2^), indicating enhanced charge transfer kinetics in PCN-ZnO_0.4_ owing to improved electrochemical charge transport.^44,45^

**Figure 7:**
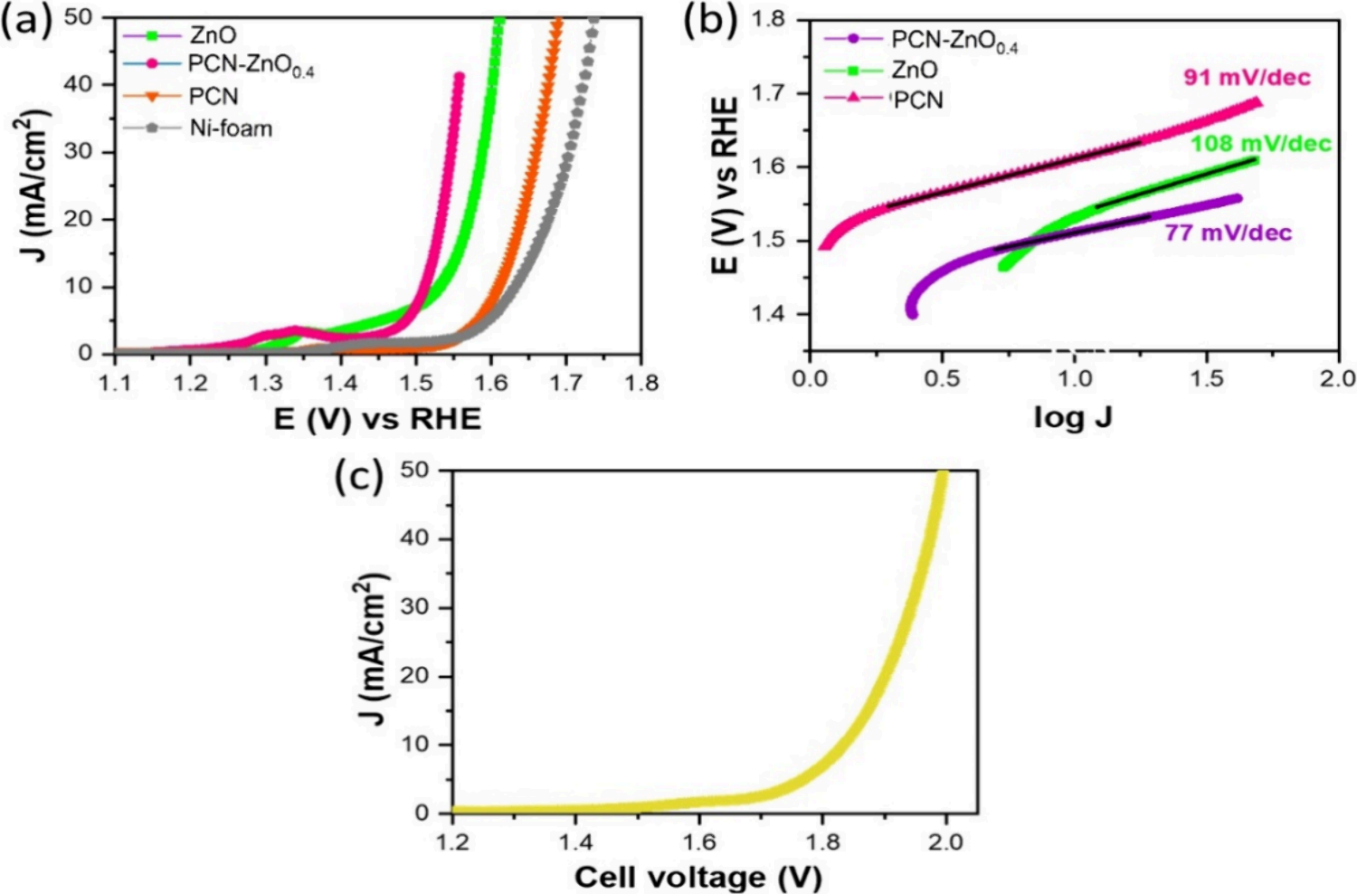
Oxygen evolution reaction (OER) on ZnO, PCN, PCN-ZnO0.4, and bare NF electrocatalyst. (a) Polarization curves recorded at a scan rate of 5 mV/s with *iR* compensation, (b) Tafel plots for OER, (c) and polarization curves recorded at a scan rate of 5 mV/s for bifunctional water splitting using a two-electrode electrolyzer containing PCNznO0.4 as the anode and cathode.

We further investigated the electrocatalytic OER performance of ZnO, PCN, PCN-ZnO_0.4_, and the bare NF electrocatalyst in 1 M KOH. Figure 7 a shows the polarization curve for OER, showing that PCN-ZnO_0.4_ generates 10 mA/cm^2^ current density at the potential of 1.511 V vs RHE, which is low compared to the potential required for the control electrodes based on pristine ZnO (1.532 V vs RHE) and PCN (1.612 V vs RHE).

The overpotential for the PCN-ZnO_0.4_ interfaced electrocatalyst is 281 mV, which is significantly smaller than that of pristine ZnO (302 mV) and PCN (382 mV). The OER performance of PCN-ZnO_0.4_ is superior to previous reports owing to the synergy between multimetals and the enhanced electrocatalytic surface area. Tafel slope values of 108, 91, and 77 mV/dec are observed for pristine ZnO, pristine PCN, and PCN-ZnO_0.4_ interfaced electrocatalyst, respectively (Figure 7b). After the confirmation of accelerated HER and OER activities on the PCN-ZnO_0.4_ interfaced electrocatalyst, a two-electrode alkaline electrolyzer containing PCN-ZnO_0.4_ electrodes as both anode and cathode were further analyzed for bifunctional electrolysis for simultaneous H_2_ and O_2_ production. Figure 7c shows the polarization curve for the bifunctional electrolysis, showing that the cell voltage of 1.83 V generates a geometric current density of 10 mA/cm^2^.

### 3.8 Density Functional Theory (DFT) Calculation

To comprehend the sole reason for the increased catalytic activity following heterostructure formation, we performed hydrogen evolution reaction and oxygen evolution reaction mechanisms for both the pristine polymeric carbon nitride monolayer and PCN-ZnO heterostructure.

**Figure 8:**
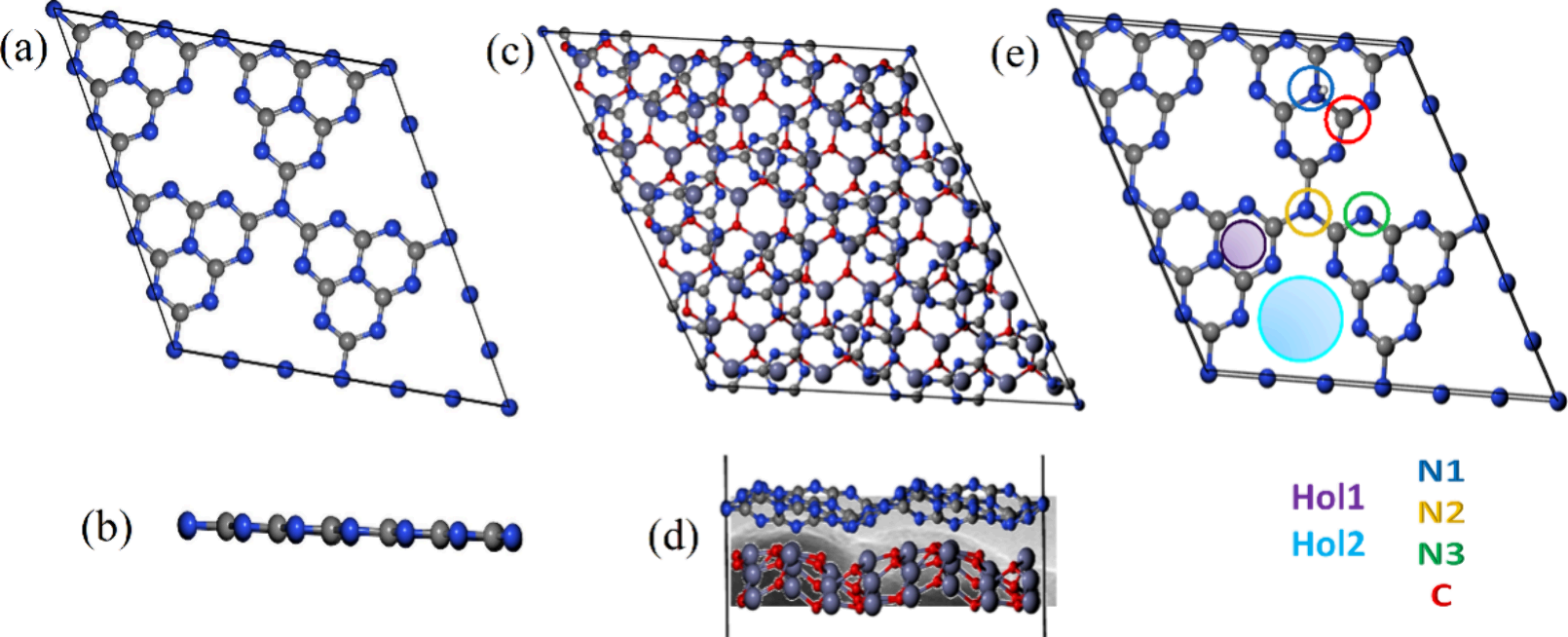
(a) Top and (b) side view of PCN, (c) top and (d) side view (along with a snapshot of the TEM image in the background as reference) of PCN-ZnO heterostructure together with a snapshot of the TEM of the nanocomposite, and (e) sites for Volmer–hydrogen adsorption on the PCN monolayer, where Hol1 and Hol1 are hollow sites; N1, N2, and N3 are the top sites of the nitrogen atom; and C is the top site of the carbon atom of the PCN monolayer, respectively.

Here, we investigated the catalytic properties alongside the structural and electronic properties to corroborate the experimental outcomes. To the best of our knowledge, investigations into the PCN-ZnO heterostructure using the density functional theory have yet to be conducted. Initially, to construct the heterostructure, we optimized individual monolayers of PCN and ZnO with the (001) slab of bulk p-C_3_N_4_ and wurtzite ZnO.^46,47^ The lattice constants of a unit cell for the PCN and ZnO monolayers were found to be 3.26 and 7.11 Å, respectively, which are consistent with previous reports.^47,48^ Subsequently, to minimize the lattice mismatch between the two pristine monolayers, we optimized a √3 × √3 supercell for PCN and a 4 × 4 supercell for ZnO. We introduced a distance of 30 Å between two periodic images of the pristine monolayer, while for the heterostructure, this distance was increased to 40 Å to prevent interaction between them. The optimized structures of the supercells for the PCN monolayer and PCN-ZnO heterostructure are depicted in Figure 8. Following the optimization of the PCN-ZnO heterostructure, we observed that the structure resembles the TEM of the nanocomposites. This inspired us to investigate its catalytic activity for complete water splitting, focusing on the Volmer–Heyrovsky and Volmer–Tafel mechanisms to determine the dominant hydrogen evolution reaction mechanism.

The band gaps obtained using the PBE functional for all three considered systems are found to be 0.68, 1.23, and 1.68 eV for the PCN-ZnO heterostructure (Figure S5), PCN, and ZnO monolayer (ML), respectively. It is well-known that the PBE function tends to underestimate the band gap. However, the HSE06 method provides a more accurate estimation closer to experimental observations without altering the nature of the bands obtained from the PBE function. Therefore, we calculated the band gap using the HSE06 hybrid functional. The resulting electronic band gaps are 1.85, 2.67, and 3.1 eV, respectively, for the PCN-ZnO heterostructure (Hs), PCN ML, and ZnO ML.

Here, we employed the computational hydrogen electrode (CHE) approach^21,22^ to evaluate the hydrogen evolution reaction (HER) and oxygen evolution reaction (OER). Initially, we analyzed the Volmer reaction with the absorption of H^+^ at various sites, as illustrated in Figure 8c. We assessed the adsorption energy and Volmer–Gibbs free energy dependence on pH, as described in equations elsewhere.^47,49,50^ The obtained change in zero-point energy for PCN and PCN-ZnO heterostructure is approximately 0.15 and 0.06 eV, respectively. The corresponding ΔZPE – *T*Δ*S* values are 0.35 and 0.26 eV, respectively, for PCN ML^49^ and PCN-ZnO HS. We observed that hydrogen strongly bonds at the C position with an adsorption energy of −1.947 eV, whereas the lowest adsorption energy is found at the middle nitrogen (N1 site) of heptazine, approximately −0.148. According to the Sabatier principle, if hydrogen absorbs too strongly, it may hinder the evolution of H_2_.^51^ Therefore, nitrogen sites are more favorable for the evolution of H_2_ in the case of PCN ML, which is consistent with previous reports.^47,49^ The preferred position for Volmer–Hydrogen adsorption for PCN-ZnO HS is the carbon site, as it exhibits the lowest adsorption energy (−0.161 eV). The primary reason for this favorable site is the interaction between the nitrogen atoms of PCN and the zinc atoms of the ZnO layer in the PCN-ZnO HS, resulting in positive adsorption energies with values of 0.91, 1.11, and −0.97 eV, respectively, for N1, N2, and N3. Interestingly, the hollow position exhibits lower adsorption energies than the pristine ML, which are approximately −0.66 and −0.93 eV, respectively, for Hol_1_ and Hol_2_. The Volmer–Gibbs free energy is 0.099 and 0.202 eV for PCN-ZnO HS and PCN ML, respectively. HS exhibits a lower Gibbs free energy. Therefore, we claim that it shows a higher catalytic HER activity. The corresponding overpotential (η = |Δ*G*|/*e*) is 99 and 202 mV for PCN-ZnO HS and PCN ML, which is consistent with our experimental.

To confirm that the interaction as well as the charge transfer mechanism leads to better adsorption performance, we conducted a Lowdin charge analysis on the best results obtained. We observed low charge transfer in PCN-ZnO HS from Volmer-hydrogen compared to PCN ML. Moreover, we noticed a charge transfer from ZnO to PCN, approximately 0.02 e, indicating a strong donor–acceptor interface. The charge transfer from hydrogen to PCN is about 0.06 e for PCN-ZnO HS, whereas it is 0.19 e from hydrogen to pristine PCN ML. Therefore, the charge redistribution at the interface plays a vital role in the adsorption of Volmer-hydrogen at site C compared with site N.

Further, we considered the entire HER mechanism and performed the adsorption of a second hydrogen on top of the Volmer-hydrogen, known as the Volmer–Heyrovsky mechanism. We observed that the evolution of H_2_ is possible with the Volmer–Heyrovsky path reaction in both cases.

The calculated Volmer–Heyrovsky Gibbs free energy for PCN-ZnO Hs and PCN ML is −0.043 and −0.136 eV, respectively. This confirms that HS exhibits better catalytic activity and a lower kinetic barrier than PCN ML.

**Figure 9:**
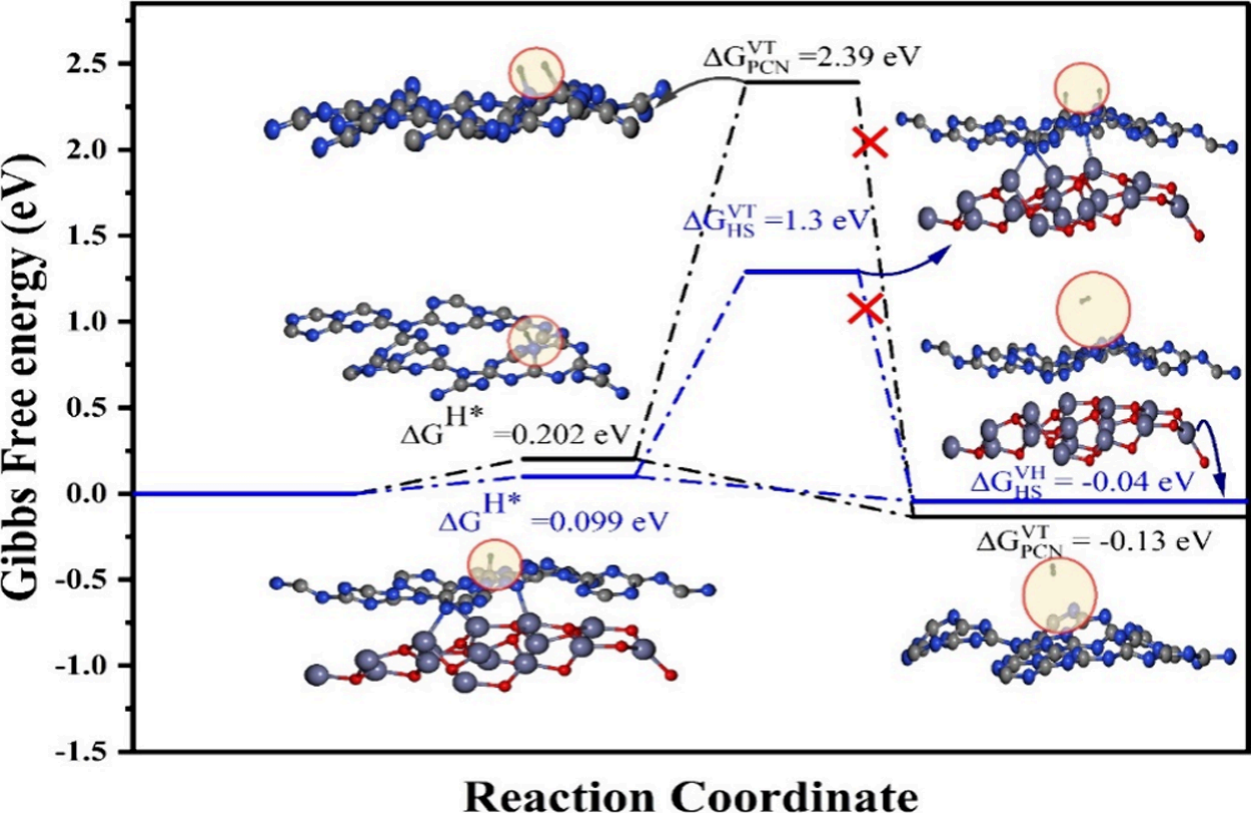
Hydrogen evolution reaction coordination path of PCN and the PCN-ZnO heterostructure.

In the case of the Volmer–Tafel mechanism, we observed no evolution of H_2_ gas, as the second hydrogen (say Tafel hydrogen) strongly adsorbs to the carbon site, causing both Volmer and Tafel hydrogen to separate from each other in both cases (see Figure 9) of pristine PCN and PCN-ZnO HS. In summary, we observed that the Volmer–Heyrovsky mechanism is solely responsible for the evolution of H_2_ gas, validating our experimental observations.

After that, we investigated the OER, with adsorption of the intermediates O*, OH*, and OOH* in both PCN-ZnO HS and PCN ML. The favorable sites for the adsorption of these intermediates are N3 (0.27 eV), N3 (0.44 eV), and N1 (4.31 eV), respectively. Because OOH* intermediates react with H_2_O molecules, leading to the evolution of O_2_ gas, oxygen will evolve at the N1 site, whereas hydrogen is at the C site. Both reactions can occur simultaneously on the PCN-ZnO HS, demonstrating bifunctioning electrolysis. The calculated overpotential using the equations^47^ is 1.84 V. Similarly, we investigated PCN; the preferable site for the evolution of O_2_ is the C site, having an overpotential of about 2.13 V, which is higher than the PCN-ZnO HS. In both the HER and OER, PCN-ZnO HS shows better catalytic activity.

The photocatalytic activity of the heterostructure and PCN monolayer depends on several factors, including the position of the maximum valence band and minimum conduction band, recombination rate, and solar-to-hydrogen efficiency. The recombination rate can be estimated from the ratio of the effective mass of the charge carriers. The calculated effective mass for the hole _(_*m*_h_^*^_)_ and electron _(_*m*_e_^*^_)_ for pristine PCN ML along the K-Γ path is 0.92 and 0.74 *m*_e_, and their active ratio (*D* = *m*_h_^*^/*m*_e_^*^) is 1.27. In the case of the PCN-ZnO HS, the effective mass for hole and electron along the K-path is 2.3 and 1.49 *m*_e_, and *D* is 1.54. Here, the HS exhibits higher effective mass, which is associated with longer carrier lifetimes and improved charge separation efficiency. This phenomenon was previously attributed to slower recombination rates.^4,46^ We calculated the solar to hydrogen efficiency (STH) as described in the work of Wang et al. The STH of PCN ML is 5.18%, which is consistent with their report.^47^ We observed that the STH of PCN-ZnO is four times more (21.75%) than that of PCN ML.

In summary, our findings demonstrate that direct calcination of melamine to form PCN-ZnO nanocomposites is notably more effective than liquid exfoliation and thermal oxidation etching methods in tuning and reducing the band gap, thereby enhancing hydrogen production. The PCN-ZnO_0.4_ nanocomposite shows superior catalytic activity for water splitting with an overpotential of 281 mV, significantly lower than those of pristine ZnO and PCN. This research underscores the potential of PCN-ZnO nanocomposites as highly efficient electrocatalysts for hydrogen generation. The presence of the RS ZnO phase provides higher stability to the PCN nanocomposite compared to the PZ nanocomposite. DFT demonstrates that PCN-ZnO HS acquires longer carrier lifetimes and improved charge separation efficiency, leading to a slower recombination rate and enhanced solar-to-hydrogen conversion efficiency.

## 4 Conclusions

Our findings confirm the development of PCN-ZnO nanocomposites using melamine precursors, instead of the conventional urea-based methods, leads to significant improvements in structural, stability, and catalytic properties for overall water splitting. It was also found that the properties of the nanocomposite produced with melamine as a precursor depend strongly on the synthesis methods. Thermal condensation (Method 1) leads to a reduction in the band gap and a lower specific surface area, while liquid exfoliation (Method 2) results in an increased surface area with only a slight change in the band gap.

Regarding catalytic properties, it was demonstrated for the first time that the PCN-ZnO_0.4_ nanocomposite can serve as a bifunctional electrocatalyst for water splitting, capable of producing both hydrogen (H__2__) and oxygen (O__2__) in alkaline electrolytes. It exhibits enhanced oxygen evolution reaction (OER) performance, making it promising for simultaneous H__2__ and O__2__ production. The PCN-ZnO_0.4_ nanocomposite synthesized via Method 1 shows an overpotential of 281 mV for HER, significantly lower than those of pristine PCN (382 mV) and ZnO (302 mV).

Comparing experimental data with the results of density functional theory (DFT) calculations allows us to explain the hydrogen evolution reaction (HER) mechanism and the enhanced catalytic activity due to the charge transfer from ZnO to PCN monolayer. DFT calculations confirm hydrogen evolution through the Heyrovsky process, in strong alignment with experimental findings, providing a comprehensive understanding of the reaction mechanism and the role of active sites in the PCN-ZnO heterostructure (HS). The calculated solar-to-hydrogen (STH) efficiency of the PCN-ZnO HS is four times higher (21.75%) compared to that of the PCN monolayer (5.18%), highlighting its potential for efficient solar-driven hydrogen production.

In summary, our research underscores the potential of PCN-ZnO nanocomposites as exceptionally effective electrocatalysts for hydrogen generation, stressing the need for customized synthesis methodologies and comprehensive characterizations for the progression of sustainable energy technologies.

## Supporting Information Available

The synthesis details of polymeric carbon nitride and zinc oxide, characterization data including Figures S1–S7 (XRD patterns, SEM images, TGA-DSC-MS spectra, ATR-IR spectra, Tauc plots, CV curves, electronic band structures), and Tables S1–S4 (list of samples; highest intensity peak’s 2θ value, average crystallite size, density, specific surface area, Sauter mean diameter, and direct band gap; elemental atomic percentage (at. %) and C/N ratio; and comparison of HER, OER, and overall water splitting (OWS) performance of the PCN-ZnO_0.4_ nanocomposite with previous reports) (PDF)
