# Supplementary material for: Cutting-Edge PCN-ZnO Nanocomposites with Experimental and DFT Insights into Enhanced Hydrogen Evolution Reaction
Source: ACS Appl Energy Mater. 2024 Oct 11;7(20):9402–13. doi: 10.1021/acsaem.4c01932 (PMC11523037; doi:10.1021/acsaem.4c01932)
Supplement: Supplementary file 1 — ae4c01932_si_001.pdf [file ae4c01932_si_001.pdf]

Supporting Information

for

Cutting-Edge PCN-ZnO Nanocomposites with Experimental and DFT Insights into Enhanced Hydrogen Evolution Reaction

Narayan N. Som[1*], Agnieszka Opalinska[1], Madhurya Chandel[2], Pratik M. Pataniya[3], Iwona Koltsov[3], Julita Smalc-Koziorowska1, Anna Swiderska-Sroda[1], Stanislaw Gierlotka[1], Sumesh CK [3] and Witold Lojkowski[1]

[1] Institute of High Pressure Physics, Polish Academy of Science, Sokolowska 29/37, 01-142 Warsaw, Poland

[2]Faculty of Mechatronics, Warsaw University of Technology, św. Andrzeja Boboli 8, 02-525 Warsaw, Poland

[3]Department of Physical Sciences, P. D. Patel Institute of Applied Sciences, CHARUSAT,

Changa, Gujarat, India 388421

[4]Łukasiewicz Research Network, Institute of Electrical Engineering,04-703 Warszawa, Poland

*Email:somnarayan4@gmail.com

Material Synthesis

For synthesis, the following chemical were used: zinc acetate dihydrate ($Zn(CH_3COO)_2.2H_2O$, analytically pure, (SKU: 112654906-1KG, Chempur); ethylene glycol (EG, ethane-1,2- diol, $C_2H_4(OH)_2$, pure, SKU: 114466303-5L, Chempur); melamine (Sigma-Aldrich, CAS Number 108–78-1 (99%)). The usage of all chemicals was analytical grade without further purification.

Synthesis of Polymeric Carbon Nitride (PCN).

The PCN was synthesized using the direct calcination of Melamine at 550 °C for 3hrs at a heating rate of 5°C/min in a muffle furnace in air. The obtained material were grounded into powder and used as a precursor for synthesizing PCN-ZnO nanocomposite.

Synthesis of Zinc oxide using Microwave Hydrothermal Synthesis (MHS)

The synthesis of ZnO nanoparticles (NP) is described in the previous work[1]. The reaction precursor, 1500 ml of solution of zinc acetate hydrate in EG (0.3037 mol dm^{-3}), was prepared using a hotplate magnetic stirrer (SLR, SI Analytics, Germany) at the constant temperature of 70 °C, stirring speed of 250 rpm[1]. The complete dissolution of zinc acetate was transferred to a 1000 ml bottle and cooled to room temperature. Thereafter, 80 ml of solutions were transferred to 110 ml of 8 Teflon reaction containers and tightly closed. All containers were put in the ETHOS up High performance Microwave digestion system, milestone 1900W and synthesis was carried out at 220 °C for 50 min and cooled down for 20 mins. The obtained ZnO NPs were used as precursors for PCN-ZnO nanocomposite.

Characterization

The X-ray Diffraction (XRD) analysis was performed for all synthesized powder samples at room temperature in the range of 10-80° with a step size of 0.03°, on a diffractometer equipped with a copper node (Cu K 1) an ultra-fast PIXcel1D detector. FTIR-ATR characterization was carried out in the range of 500-3500 cm^{-1} using a Fourier transform infrared (FTIR)

spectrometer of Bruker Optics, Tensor 27, Bruker BioSpin GmbH, Rheinstetten, Germany. The FTIR is equipped with a diamond attenuated total reflectance (ATR) accessory, and the spectra resolution and accuracy are about 4 cm^{-1} and 1 cm^{-1} respectively. For the specific surface area, Nitrogen adsorption-desorption isotherms and the Brunauer-Emmett-Teller method were applied, using Gemini 262 2360, V 2.01, Micromeritics, Norcross, GA, USA together with Micro active Software V4.03. The morphological features of prepared samples were visually characterized by using SEM (Ultra Plus, Carl Zeiss Meditec AG, Jena, Germany) with Secondary electrons (SE) detector and TEM (FEI Talos F200X, Thermo Fisher Scientific, Waltham, MA, USA), with the beam energy of 2 keV. The thermal stability of the prepared samples was performed using the Thermogravimetric Analyzer (TGA) (STA 449 F1 Jupiter, Netzsch, Selb, Deutschland). Simultaneously, the gases released from prepared samples during the thermal treatment were analyzed using the QMS 403 C Aëolos quadrupole mass spectrometer by Netzsch. The optical properties were investigated by ultraviolet-visible diffused reflectance spectra (DRS).

(a)

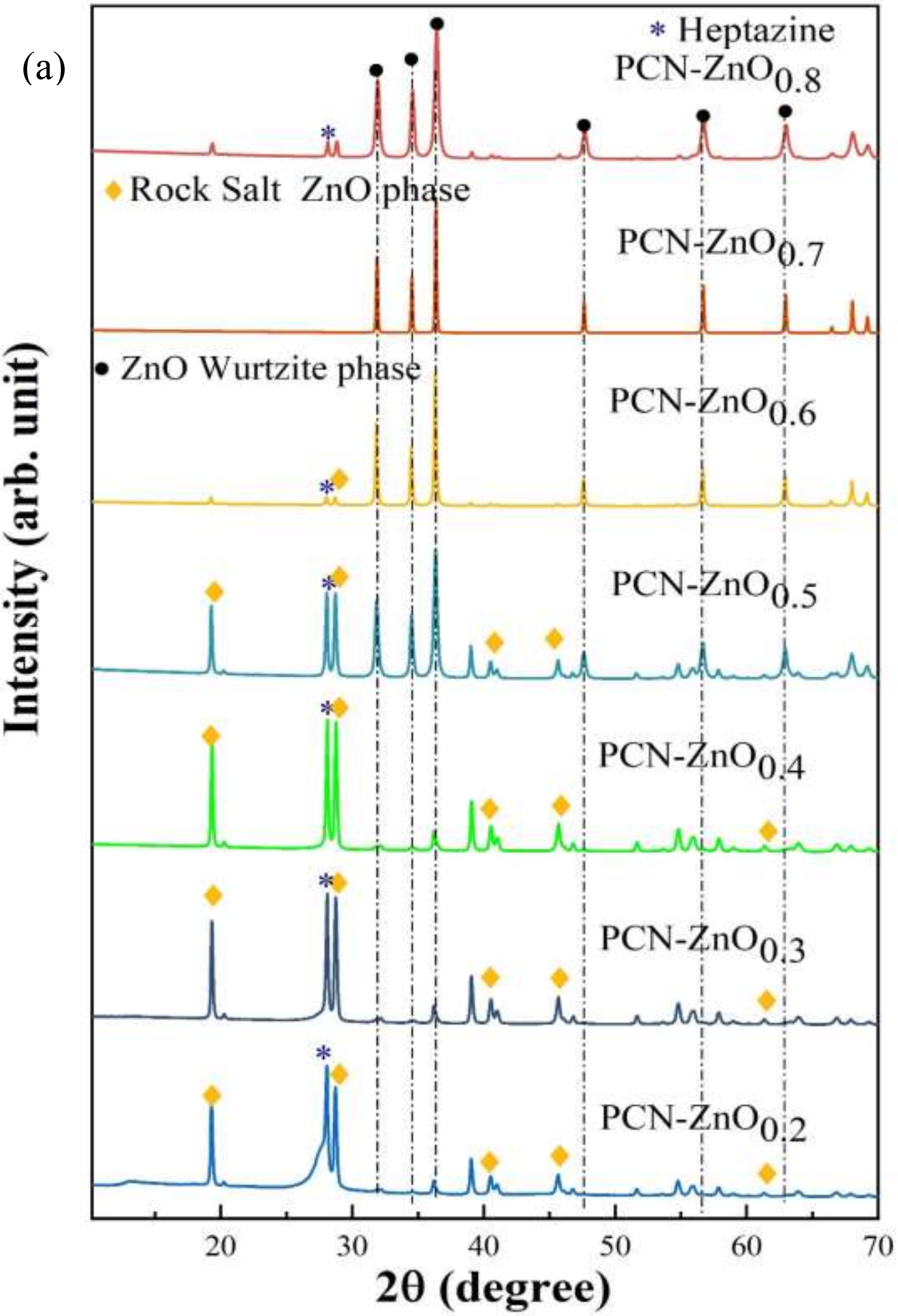

* Heptazine
PCN-ZnO0.8
◆ Rock Salt ZnO phase
PCN-ZnO0.7
● ZnO Wurtzite phase
PCN-ZnO0.6
PCN-ZnO0.5
PCN-ZnO0.4
PCN-ZnO0.3
PCN-ZnO0.2
Intensity (arb. unit)
20
30
40
50
60
70
2θ (degree)

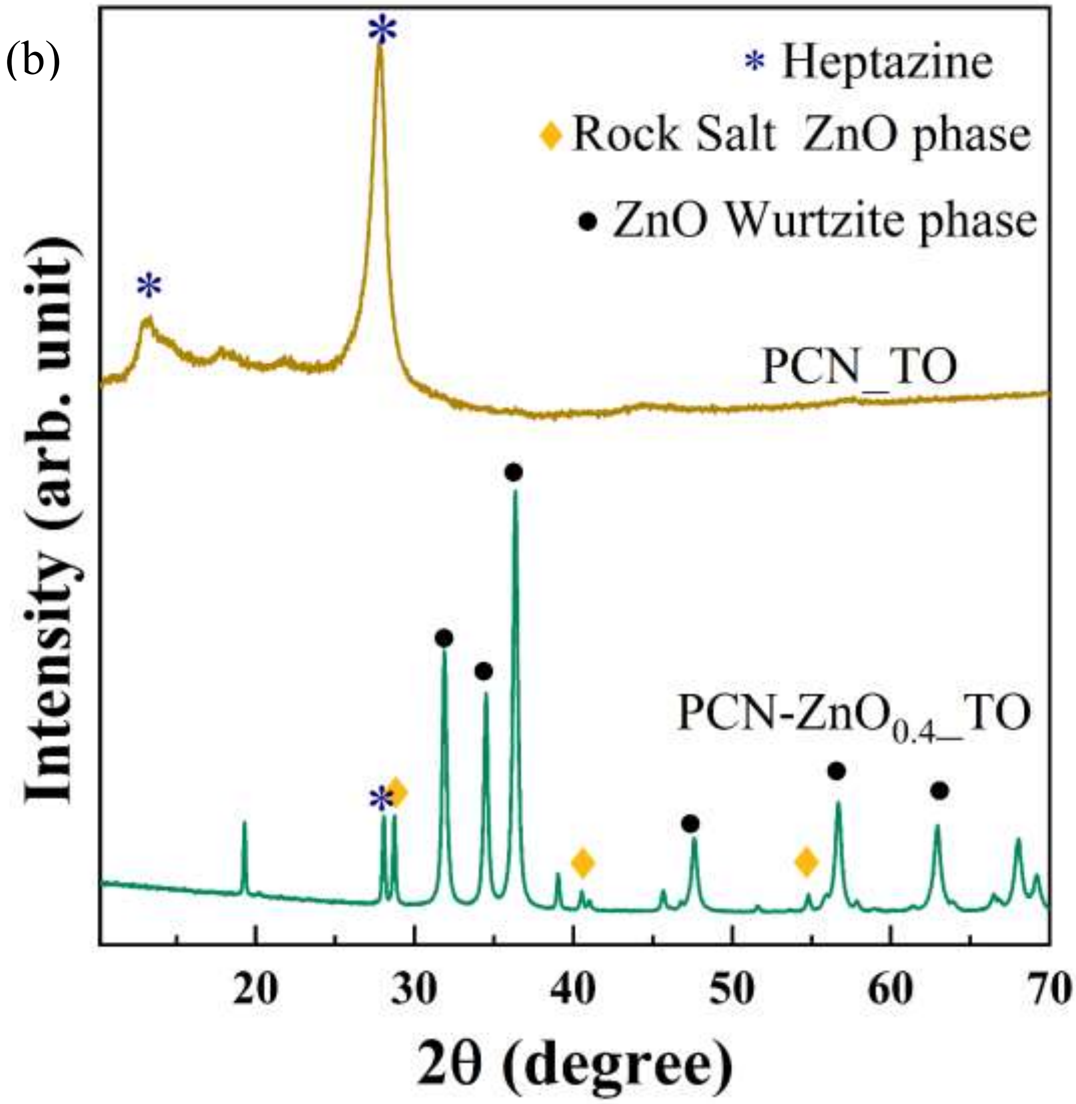

Figure S1: (a) XRD patter of PCN-ZnOx (x=0.2 to 0.8) and (b) themal oxidizing ecthing of PCN and PCN-$ZnO_{0.4}$ (say PCN_TO and PCN-$ZnO_{0.4}$_TO)

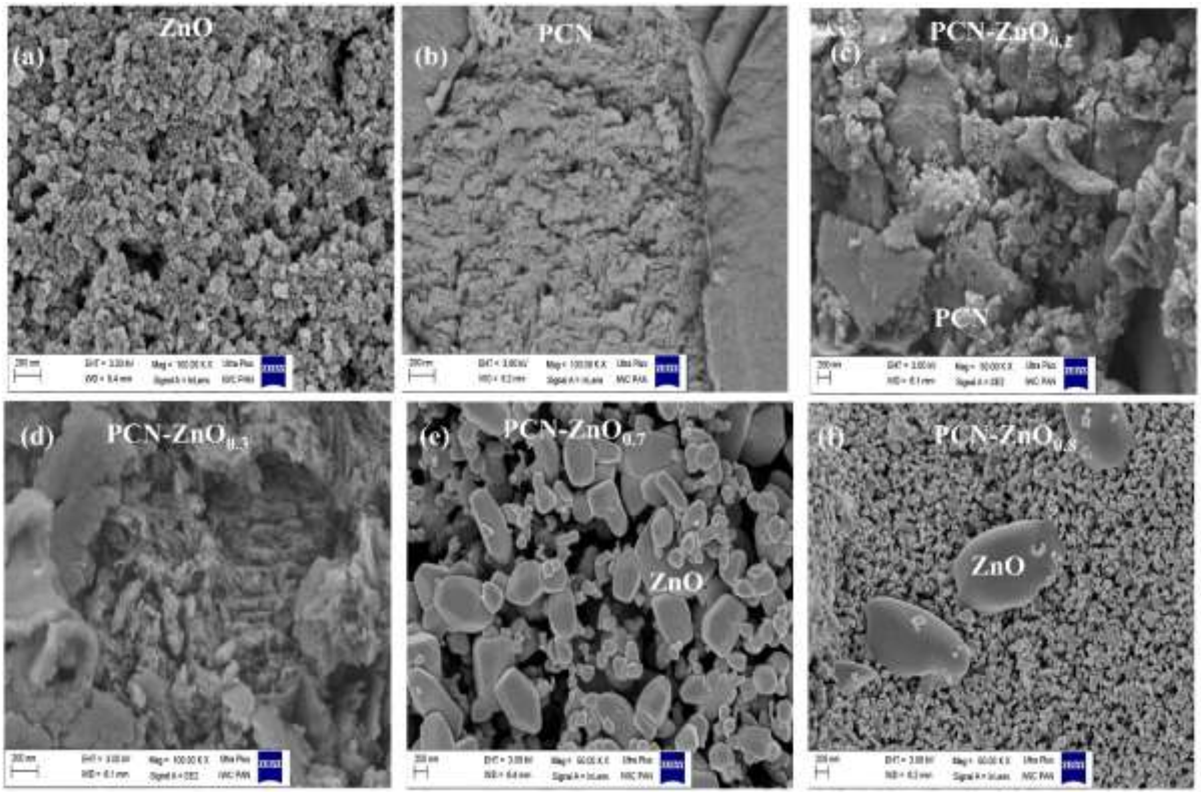

Figure S2: SEM image of (a) ZnO, (b) PCN, (c)PCN-$ZnO_{0.2}$, (d) PCN-$ZnO_{0.3}$, (e) PCN-$ZnO_{0.7}$ and (f)PCN-$ZnO_{0.8}$

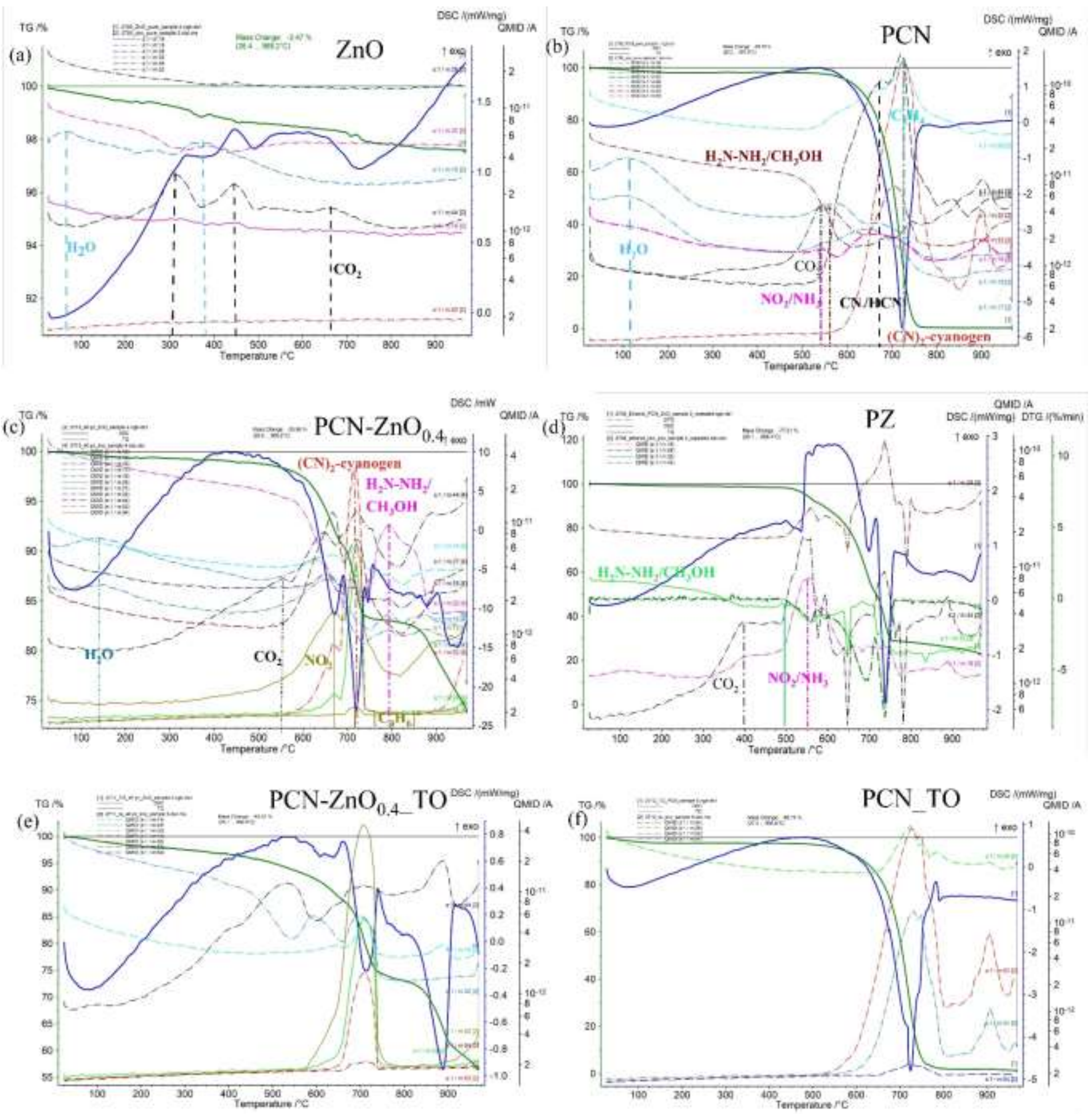

Figure S3: TGA-DSC-MS spectra for (a)ZnO, (b)PCN, (c)PCN-$ZnO_{0.4}$, (d)PZ, (e) PCN-$ZnO_{0.4}$_TO and (f) PCN_TO.

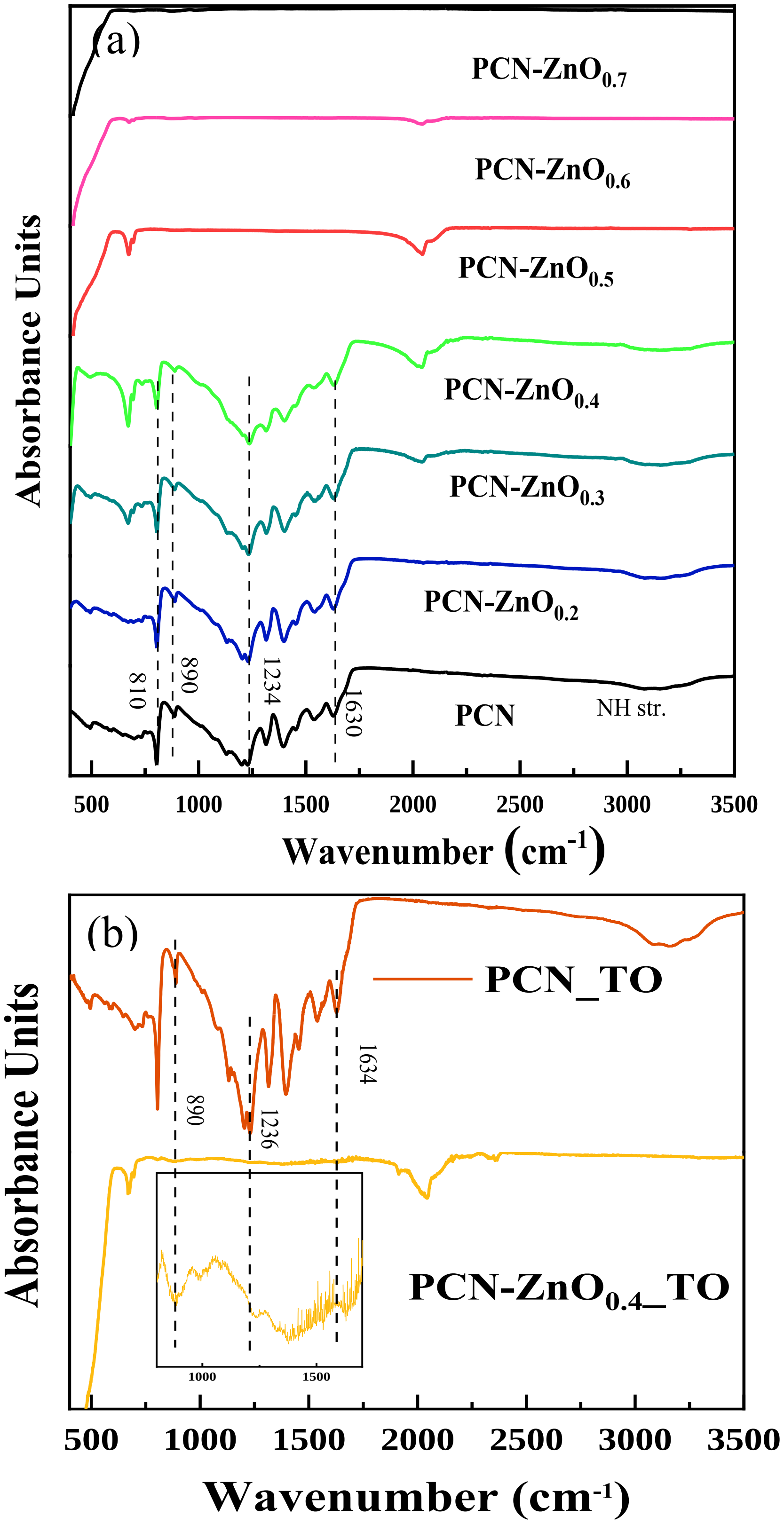

Figure S4: ATR-IR spectra for (a) PCN and PCN-ZnO_x (x= 0.2to0.7) nanocomposite and (b) PCN_TO and PCN- $ZnO_{0.4}$_TO.

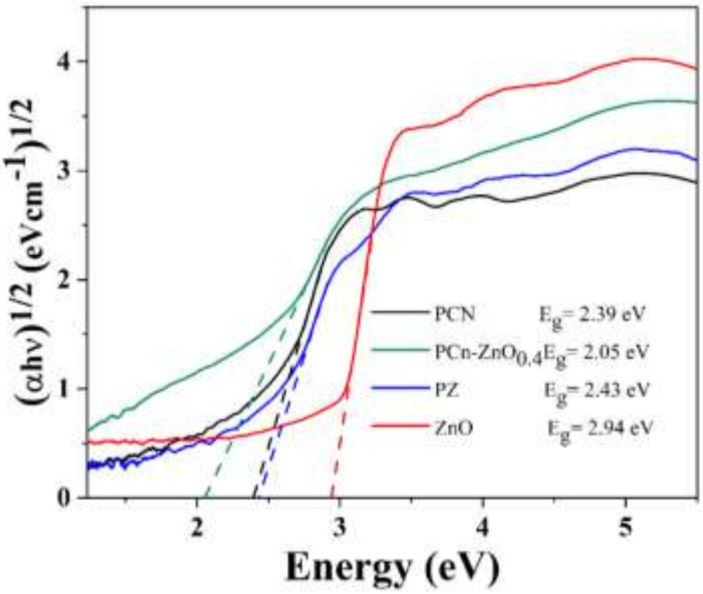

Figure S5: Tauc plot $(\alpha h\nu)^{1/2}$ Vs photon energy of PCN, ZnO, and PCN-ZnO NCs

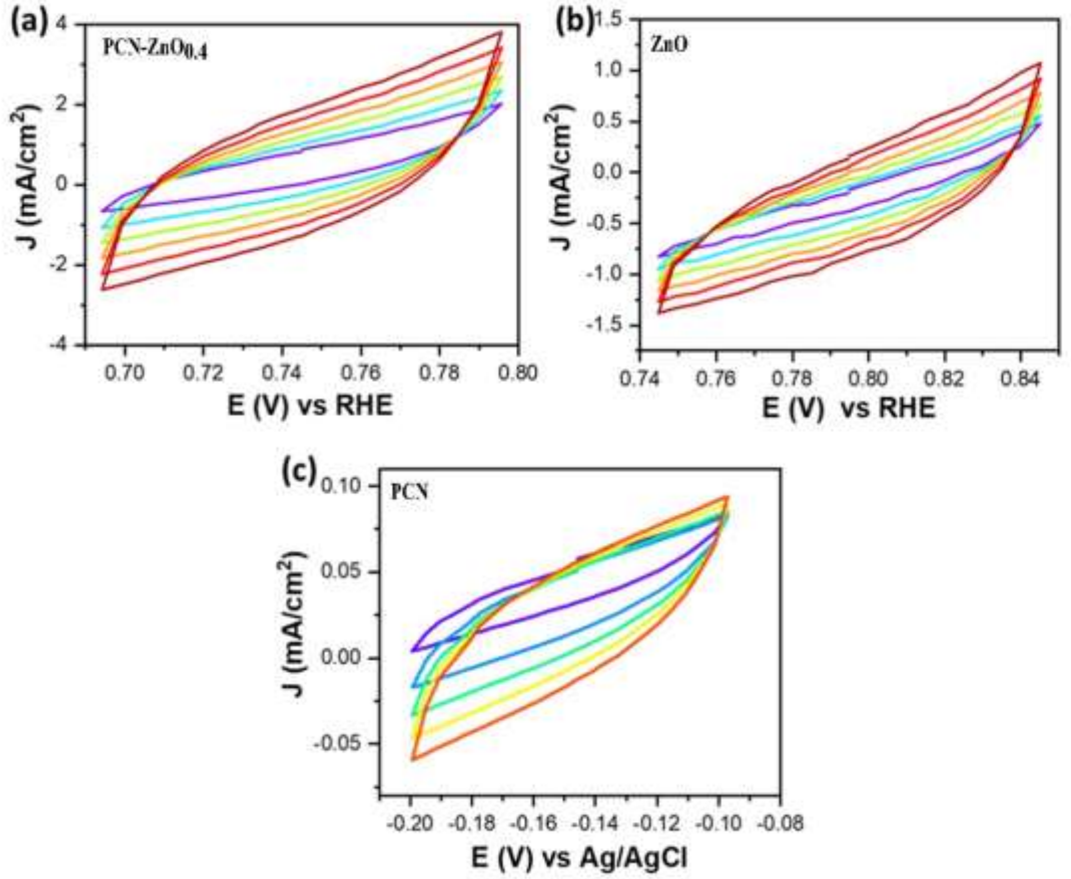

Figure S6: CV curves for (a) PCN-$ZnO_{0.4}$, (b) ZnO, and (c) PCN, electrocatalysts in non-faradic potential range at different scan rate from 10-60 mV/s.

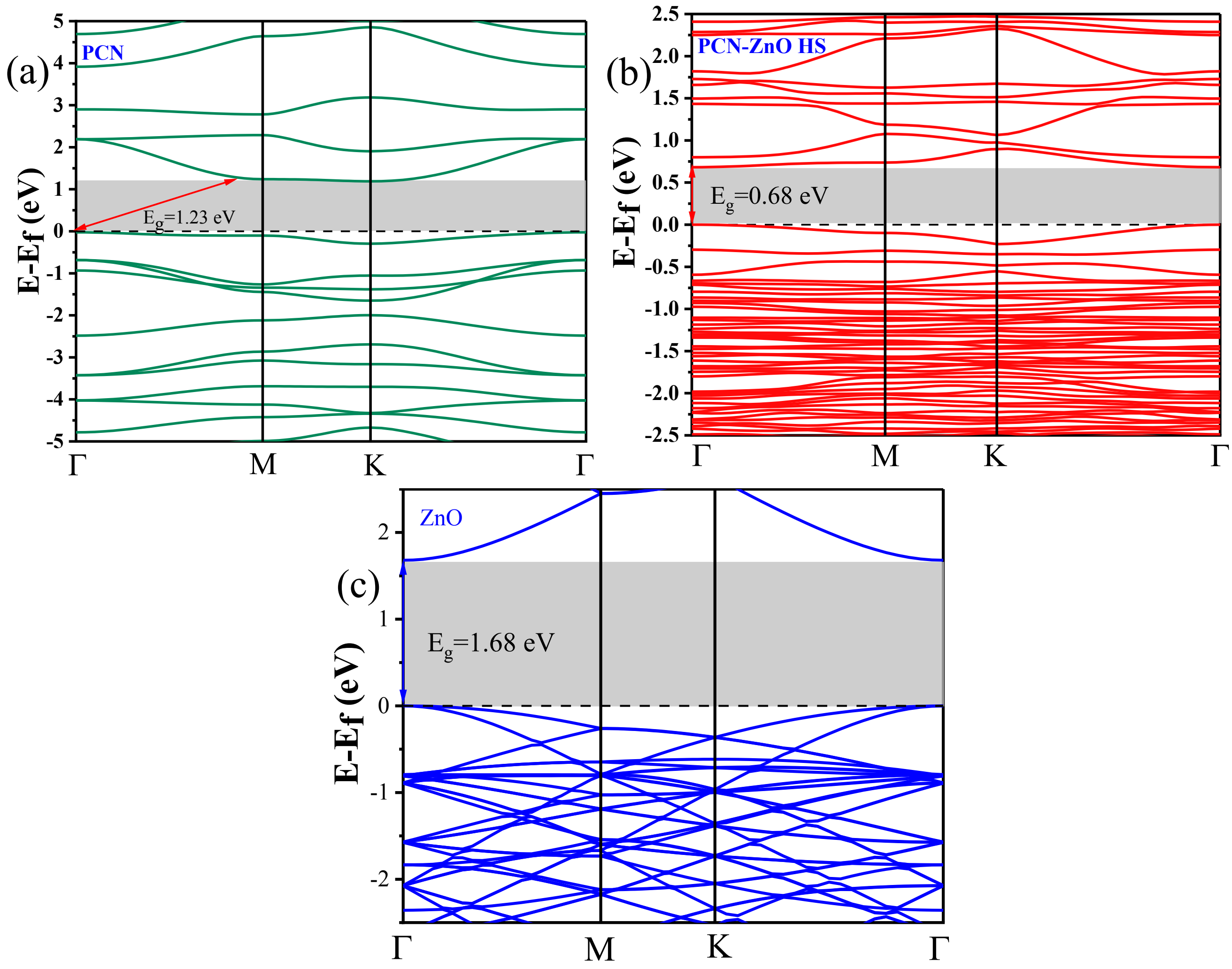

Figure S7: Electronic band structures of (a) PCN, (b) PCN-ZnO HS and (c) ZnO with PBE functional**.**

Table S1: List of the samples

Prepared Samples
ZnO
PCN
Method1
PCN-$ZnO_{0.2}$
PCN-$ZnO_{0.3}$
PCN-$ZnO_{0.4}$
PCN-$ZnO_{0.5}$
PCN-$ZnO_{0.6}$
PCN-$ZnO_{0.7}$
PCN-$ZnO_{0.8}$
Method 2
PZ (PCN/ZnO-Ethanol)
Thermal oxidation etching
40%ZnO (TO)
PCN(TO)

Table S2: Highest intensity peak's 2θ value (degree), average crystallite size (D/nm), Density (g/cm^3), Specific Surface Area (m^2/g), Sauter mean diameter (SMD/nm) and direct band gap (E_g/eV) of PCN-ZnO nanocomposites obtained from method 1.

Material	2θ	D (nm)	Density (g/cm^3)	SSA_{BET} m^2/g	SMD (nm)	Eg (eV)
PCN-$ZnO_{0.2}$	228.02	27.90	2.0261	8.864	334.09	2.55
PCN-$ZnO_{0.3}$	28.65	30.87	2.5204	4.5615	522.14	2.51
PCN-$ZnO_{0.4}$	**28.77**	**21.62**	**2.8478**	**3.115**	**676.37**	**2.44**
PCN-$ZnO_{0.5}$	36.33	35.12	4.1450	5.4029	267.92	2.89
PCN-$ZnO_{0.6}$	36.33	54.47	4.4247	7.21	188.08	3.00
PCN-$ZnO_{0.7}$	36.36	64.16	5.6824	2.2840	462.30	3.01
PCN-$ZnO_{0.8}$	36.42	28.04	5.1221	9.4607	123.88	2.96

Table S3: Elemental Atomic percentage and C/N ratio of the sample

Material	at %				Ratio
	C	N	Zn	O	C/N
PCN	39.10	57.38	-	-	0.68
ZnO	-	-	48.54	51.46	-
PCN-$ZnO_{0.2}$	41.16	55.05	1.02	2.78	0.74
PCN-$ZnO_{0.3}$	41.66	51.15	3.56	3.64	0.81
PCN-$ZnO_{0.4}$	43.45	46.54	7.00	3.01	0.93
PZ	42.56	33.04	14.93	9.46	1.29

Table S4: Comparison of HER, OER and OWS performance ZnO/C_3N_4 with previous reports.

Materials	Electrolyte	Overpotential (η_{10})	References
$NiP_2/NiSe_2$	1.0M KOH(HER)<br>1.0MKOH(OER)<br>1.0M KOH(OWS)	160mV@η_{100}<br>329mV@η_{100}<br>1.56V@η_{10}	2
$NiPS_3/Ni$	1.0M KOH (HER)<br>1.0M KOH(OER)<br>1.0M KOH(OWS)	175@η_{100}<br>273@η_{100}<br>1.6V@η_{10}	3
F-NiPx/NiS_2-NF	1.0M KOH (HER)<br>1.0M KOH(OER)<br>1.0M KOH(OWS)	182mV@η_{10}<br>370mV@η_{10}<br>1.55V@η_{10}	4
Fe-NiP	1.0M KOH (HER)<br>1.0M KOH (OER)<br>1.0M KOH (OWS)	144mV@η_{10}<br>223mV@η_{10}<br>1.59V@η_{10}	5
NC-NiFeOx@NiFe-P	1.0M KOH (HER)<br>1.0M KOH (OER)<br>1.0M KOH (OWS)	285@η_{10}<br>237@η_{10}<br>1.75@η_{10}	6
Fe_2P/Fe_4N@C	1.0M KOH (HER)<br>1.0M KOH (OER)<br>1.0M KOH (OWS)	232 @η_{10}<br>410@η_{10}<br>1.87@η_{10}	7
$Ni_xCo_{1-x}P$	1.0M KOH (HER)<br>1.0M KOH (OER)<br>1.0M KOH (OWS)	249.7 mV @η_{1000}<br>281.7mV @η_{1000}<br>1.71V@η_{500}	8
CoP/CoP_2	1.0M KOH (HER)<br>1.0M KOH (OER)<br>1.0M KOH (OWS)	239@η_{10}<br>250@η_{10}<br>-	9
$CoP/CoCr_2O_4$	1.0M KOH (HER)<br>1.0M KOH (OER)<br>1.0M KOH (OWS)	212@η_{10}<br>290@η_{10}<br>1.73@η_{10}	10
CNTs@NiP_2/NbP	1.0M KOH (HER)<br>1.0M KOH (OER)<br>1.0M KOH (OWS)	247.0 mV@η_{50}<br>274.1 mV@η_{50}<br>2.32 V@η_{100}	11
CoP(MoP)-CoMoO3@CN	1.0M KOH (HER)<br>1.0M KOH (OER)<br>1.0M KOH (OWS)	198@η_{10}<br>296@η_{10}<br>1.72@η_{10}	12
PCN-$ZnO_{0.4}$	1.0M KOH (HER)<br>1.0M KOH (OER)<br>1.0M KOH (OWS)	186@η_{10}<br>281@η_{10}<br>1.83@η_{10}	This work

References:

(1) Wojnarowicz, J.; Opalinska, A.; Chudoba, T.; Gierlotka, S.; Mukhovskyi, R.; Pietrzykowska, E.; Sobczak, K.; Lojkowski, W. Effect of Water Content in Ethylene Glycol Solvent on the Size of ZnO Nanoparticles Prepared Using Microwave Solvothermal Synthesis. *J. Nanomater.* **2016**, *2016* (2). https://doi.org/10.1155/2016/2789871.

(2) Yang, L.; Huang, L.; Yao, Y.; Jiao, L. In-Situ Construction of Lattice-Matching NiP2/NiSe2 Heterointerfaces with Electron Redistribution for Boosting Overall Water Splitting. *Appl. Catal. B Environ.* **2021**, *282*, 119584.

(3) Fang, L.; Xie, Y.; Guo, P.; Zhu, J.; Xiao, S.; Sun, S.; Zi, W.; Zhao, H. In Situ Formation of Highly Exposed NiPS 3 Nanosheets on Nickel Foam as an Efficient 3D Electrocatalyst for Overall Water Splitting. *Sustain. Energy Fuels* **2021**, *5* (9), 2537–2544.

(4) Li, K.; Tong, Y.; Feng, D.; Chen, P. Fluorine-Anion Engineering Endows Superior Bifunctional Activity of Nickel Sulfide/Phosphide Heterostructure for Overall Water Splitting. *J. Colloid Interface Sci.* **2022**, *625*, 576–584.

(5) Zhou, J.; Huang, C.; Zhou, Q.; Xie, Y.; Yang, L.; Yu, L.; Yu, Y. Electronic Structure Regulation of Nickel Phosphide for Efficient Overall Water Splitting. *Inorg. Chem.* **2022**, *61* (24), 9318–9327.

(6) Hu, Q.; Liu, X.; Tang, C.; Fan, L.; Chai, X.; Zhang, Q.; Liu, J.; He, C. Facile Fabrication of a 3D Network Composed of N-Doped Carbon-Coated Core–Shell Metal Oxides/Phosphides for Highly Efficient Water Splitting. *Sustain. Energy Fuels* **2018**, *2* (5), 1085–1092.

(7) Fan, X.; Kong, F.; Kong, A.; Chen, A.; Zhou, Z.; Shan, Y. Covalent Porphyrin Framework-Derived Fe2P@ Fe4N-Coupled Nanoparticles Embedded in N-Doped Carbons as Efficient Trifunctional Electrocatalysts. *ACS Appl. Mater. Interfaces* **2017**, *9* (38), 32840–32850.

(8) Lv, X.; Wan, S.; Mou, T.; Han, X.; Zhang, Y.; Wang, Z.; Tao, X. Atomic- Level Surface Engineering of Nickel Phosphide Nanoarrays for Efficient Electrocatalytic Water Splitting at Large Current Density. *Adv. Funct. Mater.* **2023**, *33* (4), 2205161.

(9) Li, S.; Zhang, G.; Tu, X.; Li, J. Polycrystalline CoP/CoP2 Structures for Efficient Full Water Splitting. *ChemElectroChem* **2018**, *5* (4), 701–707.

(10) Saad, A.; Shen, H.; Cheng, Z.; Ju, Q.; Guo, H.; Munir, M.; Turak, A.; Wang, J.; Yang, M. Three-Dimensional Mesoporous Phosphide–Spinel Oxide Heterojunctions with Dual Function as Catalysts for Overall Water Splitting. *ACS Appl. Energy Mater.* **2020**, *3* (2), 1684–1693.

(11) Singh, S.; Nguyen, D. C.; Kim, N. H.; Lee, J. H. Interface Engineering Induced Electrocatalytic Behavior in Core-Shelled CNTs@NiP2/NbP Heterostructure for Highly Efficient Overall Water Splitting. *Chem. Eng. J.* **2022**, *442*. https://doi.org/10.1016/j.cej.2022.136120.

(12) Yu, L.; Xiao, Y.; Luan, C.; Yang, J.; Qiao, H.; Wang, Y.; Zhang, X.; Dai, X.; Yang, Y.; Zhao, H. Cobalt/Molybdenum Phosphide and Oxide Heterostructures Encapsulated in N-Doped Carbon Nanocomposite for Overall Water Splitting in Alkaline Media. *ACS Appl. Mater. Interfaces* **2019**, *11* (7), 6890–6899.
